# Delivering a complex mental health intervention in low-resource settings: lessons from the implementation of the PRIME mental healthcare plan in primary care in Sehore district, Madhya Pradesh, India

**DOI:** 10.1192/bjo.2019.53

**Published:** 2019-07-29

**Authors:** Rahul Shidhaye, Vaibhav Murhar, Shital Muke, Ritu Shrivastava, Azaz Khan, Abhishek Singh, Erica Breuer

**Affiliations:** Senior Research Scientist and Associate Professor, Center for Chronic Conditions and Injuries, Public Health Foundation of India, India; Project Director, PRIME, Sangath, India; Research Coordinator, PRIME, Sangath, India; Researcher, PRIME, Sangath, India; Intervention Coordinator, PRIME, Sangath, India; Alan J Flisher Centre for Public Mental Health, University of Cape Town, South Africa; and Conjoint Lecturer, University of Newcastle, Australia

**Keywords:** Primary care, low and middle income countries, alcohol disorders, depressive disorders

## Abstract

**Background:**

The PRogramme for Improving Mental health care (PRIME) designed, implemented and evaluated a comprehensive mental healthcare plan (MHCP) for Sehore district, Madhya Pradesh, India.

**Aims:**

To provide quantitative measures of outputs related to implementation processes, describe the role of contextual factors that facilitated and impeded implementation processes, and discuss what has been learned from the MHCP implementation.

**Method:**

A convergent parallel mixed-methods design was used. The quantitative strand consisted of process data on mental health indicators whereas the qualitative strand consisted of in-depth interviews and focus group discussions with key stakeholders involved in PRIME implementation.

**Results:**

The implementation of the MHCP in Sehore district in Madhya Pradesh, India, demonstrated that it is feasible to establish structures (for example Mann-Kaksha) and operationalise processes to integrate mental health services in a ‘real-world’ low-resource primary care setting. The key lessons can be summarised as: (a) clear ‘process maps’ of clinical interventions and implementation steps are helpful in monitoring/tracking the progress; (b) implementation support from an external team, in addition to training of service providers, is essential to provide clinical supervision and address the implementation barriers; (c) the enabling packages of the MHCP play a crucial role in strengthening the health system and improving the context/settings for implementation; and (d) engagement with key community stakeholders and incentives for community health workers are necessary to deliver services at the community-platform level.

**Conclusions:**

The PRIME implementation model could be used to scale-up mental health services across India and similar low-resource settings.

**Declaration of interest:**

None.

In India, access to mental healthcare is poor because of multiple demand and supply side barriers. This has led to a large treatment gap for both common mental disorders (85%) and alcohol use disorder (AUD) (86.3%) according to the National Mental Health Survey.^1^ The new Mental Health Care Act 2017 provides a unique opportunity to bridge this treatment gap in India.^2^ However, there are very few evidence-based implementation models in India that provide a feasible plan to integrate mental healthcare delivery in the existing primary healthcare system in order to improve access to care. The PRogramme for Improving Mental health carE (PRIME) is an implementation research project that aimed to address this important research gap in Ethiopia, India, Nepal, South Africa and Uganda.^3^ In India, a district mental healthcare plan (MHCP) was developed for implementation in the Sehore district in the State of Madhya Pradesh,^4^ the details of which are provided elsewhere.^5^ A comprehensive, cross-country evaluation protocol was designed to assess the impact of the PRIME MHCP.^6^ The findings of the impact evaluation (reported elsewhere) demonstrate moderate improvement in patient-level outcomes (depression and AUD symptom severity), minimal improvement in detection of depression and AUD in health facilities and no change in contact coverage at the community level.^7^ Nevertheless, there are important learnings from the MHCP implementation that can be used to design appropriate implementation science studies as well as future scale-up of mental health services in the State of Madhya Pradesh and other low-resource settings across the globe.

In this paper, we describe the findings of the mixed-methods evaluation of PRIME MHCP implementation in India. The aims of this paper are: (a) to provide quantitative measures of outputs related to implementation processes; (b) to describe the role of contextual factors that facilitated and impeded implementation processes; and (c) to discuss what has been learned from the MHCP implementation.

## Method

### Setting

In India, PRIME was implemented in the Sehore district in the State of Madhya Pradesh. It has a population of 1.3 million, which is predominantly rural (81%) and the district covers an area of 6578 km^2^. Situational analysis identified a range of major health system challenges in developing and implementing the MHCP in Sehore district. Poor governance, lack of sustainable mental health financing measures, severe shortages of skilled mental health professionals in the public health system, very few capacity-building initiatives for general health professionals and non-specialists health workers, non-availability of essential psychotropic drugs, underdeveloped mental health information systems, and low mental health literacy and stigma against people with mental disorders were some of the major implementation challenges.^5^

### PRIME MHCP

The PRIME MHCP was developed using a thorough situational analysis to understand the local context, theory of change workshops to map the outcomes framework for integration of mental health in primary care, a qualitative study with key stakeholders and pilot implementation in one community health centre (CHC).^4^ Theory of change is a comprehensive description and illustration of how and why a desired change is expected to happen in a particular context. Theory of change tries to map out the link between programme activities and interventions, the outputs and outcomes and the desired goal of the programme.^8,9^ MHCP development was done in partnership with the Department of Health Services, Government of Madhya Pradesh and other key stakeholders. The PRIME MHCP was then implemented in three CHCs in Sehore district for a period of 25 months from 1 August 2014 until 31 August 2016. In Madhya Pradesh, each district has one district hospital located at the district headquarters and at the next level there are CHCs. A CHC caters to a population of around 150 000 in Madhya Pradesh. The three CHCs where PRIME was implemented are situated in towns, but mostly serve the rural population and on average there are 1600 out-patients (including adults and children) per month.

The PRIME MHCP consisted of service delivery and enabling packages. The three enabling packages comprised of cross-cutting interventions to ensure the smooth implementation of core mental health service delivery packages were: (a) programme management; (b) capacity building; and (c) community mobilisation.

The service delivery packages were: (a) awareness creation; (b) detection; (c) treatment; and (d) follow-up for three priority mental disorders: depression (including maternal depression), psychosis and AUD. The key implementation steps for each of these packages are described in an earlier publication.^4^

Mental health services were delivered on both healthcare and community platforms at the CHC and village level, respectively. The mental health case managers employed by PRIME played a very important role in implementation of the MHCP. At the healthcare-platform level, they coordinated provision of mental health services in ‘Mann-Kaksha’, a room in each CHC that was allocated for the mental health programme. At the community-platform level, the case managers visited villages that were in the catchment areas of their respective CHCs to provide services.

### MHCP service delivery packages

#### Healthcare platform

- *Detection*: case managers contacted patients attending out-patient clinics and if their presenting complaints were similar to those mentioned in the master chart of the Mental Health Gap Action Programme (mhGAP) Intervention Guide,^10^ they screened patients using Primary Health Questionnaire-9 (PHQ-9)^11,12^ for depression and Alcohol Use Disorder Identification Test (AUDIT).^13,14^ Case managers shared the PHQ-9/AUDIT scores with the medical officer who made the diagnosis. Patients with psychosis were primarily identified by case managers in the community (see below).
- *Treatment*: patients who received a confirmed diagnosis of depression or AUD by the medical officer were offered manualised counselling interventions by the case managers. For depression, this was the Healthy Activity Programme (HAP), based on behavioural activation that includes (i) psychoeducation, (ii) behavioural assessment, (iv) activity monitoring, (v) activity structuring and scheduling, (vi) activation of social networks and (vii) problem-solving.^12^ For alcohol use this was Counselling for Alcohol Problems (CAP) based on motivational interviewing.^15^ Women attending antenatal/postnatal clinics were also screened by case managers and if found positive for depression, they were offered HAP. HAP and CAP were delivered by the case managers. Depending on the severity of the disorders, the medical officers took the decision to prescribe antidepressants/other medications to patients with depression/AUD. For patients with psychosis (and their caregivers), case managers provided psychoeducation and facilitated consultation with the psychiatrist. (b)   The district mental health programme psychiatrist visited each of the CHC once a month to provide consultation for severe cases, especially those with psychosis.
- *Follow-up*: case managers maintained regular follow-up with enrolled patients.

#### Community platform

- *Detection*: during their visit to a village, case managers met the community health workers in that village (for example accredited social health activists (ASHAs)) and based on their inputs contacted individuals to assess them for priority mental disorders using the master chart in the mhGAP Intervention Guide.
- *Treatment*: case managers provided mental health first aid (MHFA) to these individuals and if further evaluation and management was required, a referral slip to visit the CHC was provided.
- *Follow-up*: case managers undertook home visits to follow-up with patients enrolled in the programme.

### Timeline of MHCP implementation

**Fig. 1:**
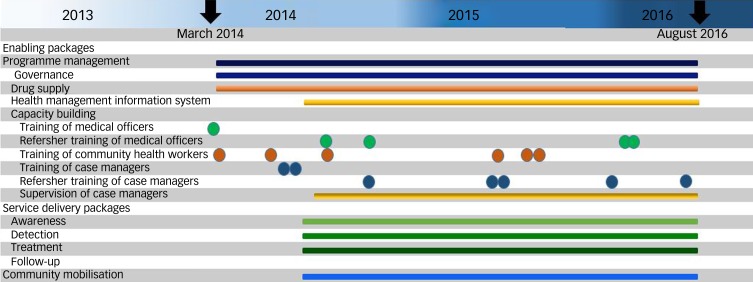
PRogramme for Improving Mental health carE (PRIME) India mental healthcare plan timelines.

Programme management activities related to governance, human resource management and drug procurement and supply chain management started in March 2014. Training of medical officers, community health workers (ASHAs) and case managers was conducted from March 2014 to July 2014. The health management information system was established in the last week of July 2014. The MHCP service delivery packages became operational in August 2014 and regular process data was collected from 1 August 2014 until the end of the implementation phase (31 August 2016). The timeline of implementation of the PRIME MHCP is depicted in Fig. 1.

### Overview of the study design

The primary outcomes of PRIME were to improve demand for mental health services at the population/community level, reduce the ‘missed opportunity’ at the health-facility level by improving detection of depression and AUD and provide evidence-based services to individuals with priority mental disorders (depression, AUD and psychosis). Our outcome evaluation was guided by the Tanahashi framework^16^ to evaluate coverage of services in public health programmes. The impact of the PRIME MHCP was assessed at three levels, namely population, health-facility and individual/patient level.^6^ The findings of the outcome evaluation mentioned above and details are reported elsewhere.^7^ The MHCP and the PRIME theory of change provided the overall framework to plan various implementation tasks as well as measure the process of implementation. The MHCP implementation was evaluated using a convergent parallel mixed-methods design. The quantitative strand of this study consisted of process data on mental health indicators related to the service delivery and enabling packages of the MHCP whereas the qualitative strand consisted of a detailed process description of the programme, in-depth interviews and focus group discussions with key stakeholders involved in PRIME implementation.

The section below separately describes the data-collection methods and analysis plan for the process evaluation and qualitative study, respectively. Tables 1 and 2 provide a summary of data-collection methods and details of the participants.

**Table 1:** Summary of data-collection methods (process evaluation)

Data collection method	Timing	Measures	Source of data
Master register (paper-based)	1 August 2014 to 31 August 2016	Sociodemographic information: age, gender, village etc Initial contact details: place of contact, presenting complaints, type of case (old/new/referral), visit number Identification and treatment details: mhGAP master chart screening result, PHQ-9/AUDIT score, diagnosis, treatment and follow-up details	Case manager filled in the details of each patient in the master register
Digital information system	1 August 2014 to 31 August 2016	Sociodemographic information: age, gender, village etc Initial contact details: place of contact, presenting complaints, type of case (old/new/referral), visit number Identification and treatment details: mhGAP master chart screening result, PHQ-9/AUDIT score, diagnosis, treatment and follow-up details	Case manager filled the details of each patient using an online form on a tablet computer that was provided to them by PRIME. This tablet was equipped with internet connection that facilitated real-time upload of data entered by case managers on a daily basis. Data manager in the PRIME team downloaded the data from the server and converted it into a Microsoft Excel spreadsheet
Profiles: district, facility and community profiles	Annually and quarterly (2014–2016)	District profile: information at district level on various domains such as available budget and trained staff, district mental health MIS, procurement system for psychotropic drug in place and other context-related events that affected the MHCP implementation Community profile: information on trained community-level staff, community volunteers and community-level activities Facility profile: information on facility characteristics such as physical structure and hours; availability of trained personnel, mental health services, medication and service utilisation and protocol/guideline availability	Data for profiles was collected by a member of the research team using semi-structured interviews of the facility managers/district officials and by review of key documents. Data was collected mostly through direct observation, the health information system, and supplemented by discussion with a facility manager and was then entered in a tablet computer using online forms
Monthly implementation log	Monthly (2014–2016)	Information on MHCP activities in a particular month, its location, objective, participants/target audience and number of people attending/reached	Implementation logs were completed on monthly basis by the programme coordinator. The completed implementation log was sent to the PRIME Research Programme Consortium

mhGAP, Mental Health Gap Action Programme; PHQ, Primary Health Questionnaire; AUDIT, Alcohol Use Disorder Identification Test; PRIME, PRogramme for Improving Mental health carE; MIS, management information system; MHCP, mental healthcare plan.

**Table 2:** Summary of data-collection methods and details of the participants (qualitative study)

Data collection method	Timing	Themes	Participants
Phase I			
In-depth interviews	April 2015 to July 2015	PRIME team: role, experience and lessons learned in designing, planning and implementing PRIME MHCP. Experience with PRIME tools, service delivery processes, information systems, building capacity of service providers and their supervision, as well as programme interaction at governance and health-system level Service providers: role in implementing MHCP Experience from training received in the programme, their experiences of working in the programme in terms of their contribution in service delivery at facility and community level, positive and negative experiences while working in PRIME/regarding service delivery and programme implementation, challenges and barriers faced during implementation, capacity building and supervision they received as well as how role of PRIME is evolving in integrating mental health services into primary healthcare and how this role could strengthen further during implementation	Service providers: In-charge CHC medical officers, *n* = 3 Community health workers , *n* = 4 District mental health programme psychiatrist, *n* = 1 PRIME team members: (total *n* = 11) PRIME core team: *n* = 5 (programme coordinator, clinical psychologist, clinical director, programme director and the principal investigator) PRIME case managers: *n* = 6
Focus group discussion (FGD)	April 2015 to July 2015	PRIME team: same as mentioned above Service providers: same as mentioned above Community advisory board: background of members, their role, frequency and number of meetings, about their interaction with the patients, PRIME research team, case managers, potential barriers and challenges and strategies or solutions to address those and also their views on scale-up of PRIME	Service providers: Community health workers: *n* = 1 FGD (*n* = 6 participants) PRIME team members: PRIME core team: *n* = 1 FGD (*n* = 7 participants) PRIME case managers: *n* = 1 FGD (*n* = 6 participants) Community advisory board: *n* = 1 FGD (*n* = 8 participants)
Phase II			
In-depth interviews	October 2016 to January 2017	Covered additional aspects that emerged from 1st phase qualitative study such as facilitator for, barriers to and challenges faced during designing, planning and implementation phases of the programme at governance, facility and community level and strategies used to address those barriers and challenges	PRIME team members: (total *n* = 11) PRIME core team: *n* = 4 (programme coordinator, clinical director, programme director and the principal investigator) PRIME case managers: *n* = 6
FGD	October 2016 to January 2017	Same as above	PRIME team members: PRIME core team: *n* = 1 FGD (*n* = 5 participants) PRIME case managers: *n* = 1 FGD (*n* = 6 participants)

PRIME, PRogramme for Improving Mental health carE; MHCP, mental healthcare plan; CHC, community health centre.

### Process evaluation

Process maps (supplementary Fig. 1, available at https://doi.org/10.1192/bjo.2019.53) were developed in August 2014 for service delivery processes in facilities. A detailed monitoring plan was developed to identify indicators associated with the implementation process. The key performance indicators were number of patients screened, PHQ-9/AUDIT scores, number of patients who received pharmacological and psychological treatment and number of follow-up sessions per patient. Sociodemographic information (age, gender, village) for each patient was also collected. A master register (paper-based) was designed to collate all the process data. Further, a digital information systems platform was developed using Mobenzi (http://mobenzi.com/researcher/home).

### Qualitative study

The qualitative study was completed in two phases. The purpose of the first phase (April 2015–July 2015) was to describe and synthesise the evolution of the procedures and processes that led to the development of the PRIME implementation model in Sehore district. The purpose of the second phase (October 2016–January 2017) was to gather additional data on process evolution after July 2015, the barriers/facilitators encountered during the implementation process and strategies designed to address these barriers.

The implementation processes related to the service delivery packages and enabling packages were first clearly described on paper by the case managers and other members of the PRIME team. These process descriptions were used to design semi-structured interview schedules for in-depth interviews and focus group discussions. The sampling of respondents for in-depth interviews and focus group discussions was purposive to ensure that the perspectives of all the stakeholders involved in the mental health service delivery and those involved in the capacity building and provision of implementation support and programme design were obtained.

### Data collection and analysis

The data pertaining to indicators (included in the process evaluation) was collected by the case managers using a tablet computer. This tablet was equipped with an internet connection that facilitated real-time upload of data entered by case managers on a daily basis. The data manager in the PRIME team downloaded the data from the server, converted it into Microsoft Excel spreadsheet and summarised the data using descriptive statistics (frequency and proportion for each indicator disaggregated by gender, disorder and facility). The summary statistics was reviewed by the programme coordinator, clinical psychologist and the programme director and was used during the weekly supervision meetings with the case managers to provide them the feedback. Case managers updated data in the master register on a daily basis and this was verified by the PRIME data manager. A monthly report was prepared based on these indicators and submitted to the in-charge medical officer in the CHC who in turn submitted it to the district health office and to the Director of Health Services, Government of Madhya Pradesh.

The in-depth interviews and focus group discussions were conducted by independent researchers in collaboration with members of the project research team. Informed consent was received from all the participants and they were also given an information sheet. Data was recorded on voice recorders and mobile devices as well as in the form of field notes by interviewers at the time of the interviews. English interviews were transcribed verbatim whereas the interviews conducted in the local language (Hindi) were transcribed and translated into English, with back-translation checks performed by the PRIME project team members fluent in the local language (Hindi).

A framework analysis approach that was specifically developed for qualitative data analysis in applied policy analysis research was used to analyse the data.^17^ The framework approach provides a systematic structure for the analysis process, with visible stages allowing funders and others to clearly follow how the results were obtained from the data.^18^ Further, it also allows for the use of *a priori* and emergent codes to be used in the analysis process.^18^ An *a priori* coding framework with a set of high-level themes was developed.

Four themes covered under the service delivery packages were: (a) awareness creation; (b) detection; (c) treatment; and (a) follow-up. Five themes covered under the enabling packages were; (a) capacity building (training and implementation support); (b) monitoring and information systems; (c) drug procurement and supply chain management; (d) human resource management; and (e) community mobilisation. For each of these nine themes, there were subthemes related to: (a) difficulties faced during setting up implementation processes; (b) barriers/facilitators in implementation; (c) strategies to address these barriers; and (d) remaining challenges. Other lower order themes were inductively derived from the data. NVivo9 software (QSR) was used to store and to code the data.

Finally, we triangulated the data from the process evaluation and the qualitative study and used the consolidated framework for implementation research (CFIR)^19^ to present our findings. We used CFIR as it is a pragmatic meta-theoretical framework that provides comprehensive taxonomy of specific constructs that help to open up the ‘black-box’ of implementation components in any public health programme. Damschroder *et al*, have proposed that CFIR can be used to organise and promote synthesis of research findings using clear and consistent language and terminology.^19^ Similar to our use of it, the majority of the studies that have used CFIR in their implementation research have used it in data-analysis phase.^20^

### Ethics

All participants gave informed consent prior to being interviewed and signed a consent form. The informed consent form made clear that there would be no negative effects for non-participation. The institutional review boards of the World Health Organization (Geneva, Switzerland), University of Cape Town (South Africa), Sangath (Goa, India) and the Indian Council of Medical Research (New Delhi, India) reviewed and approved the protocol for the study. Participants were not given any incentive to participate in the study.

## Results

The results are organised within the domains of the CFIR.

- The evidence-based interventions delivered by PRIME.
- The role of individuals in delivery and support teams, including recruitment, training, clinical supervision and implementation support delivered as part of the capacity-building and programme management packages in PRIME.
- The processes of the delivery of the MHCP service delivery packages of awareness creation and community mobilisation, detection, treatment and follow-up. In particular, we focus on the barriers/facilitators encountered during the implementation of these packages and application of implementation strategies to address these barriers.
- The impact of health systems (outer and inner setting)/contextual factors on the implementation processes and the influence of the PRIME programme management packages on these.

A summary of the results is presented in supplementary Appendix 1.

### Healthcare platform

#### Interventions

##### Source and relative advantage of evidence-based interventions

The World Health Organization's (WHO's) mhGAP Intervention Guide^10^ (for three priority disorders: depression, AUD and psychosis) and the HAP and CAP served as the evidence-based interventions for the implementation phase. The use of evidence-based interventions recommended by WHO served as a major facilitator in adoption of the intervention by decision-makers and senior health officials in the Directorate Of Health Services.

##### Adaptation

During the implementation phase, we did not undertake any major adaptations in the ‘core components’ of either the mhGAP Intervention Guide or HAP and CAP. However, we did introduce a few changes in the adaptable elements related to intervention delivery. HAP, CAP and counselling relationships manuals were translated in Hindi language and additional materials such as the session sheets, activity sheets and a drinking diary, were developed in Hindi. Contents of the psychoeducation for psychosis were taken from the Basic Needs Manual^21^ and later translated into Hindi. Certain adaptations were also made in the psychoeducation for psychosis.

##### Presentation of the intervention

In the initial days of the implementation phase, we identified the lack of an information booklet for patients as a major barrier. In order to address this, the ‘Smile Card’ was developed from the WHO Intervention Guide and HAP and CAP manuals. This small booklet (supplementary Appendix 2) contained information about the presenting symptoms of the disorder, self-care strategies that patients could use at their home, an assessment and activity tracking sheet and schedule for follow-up visits.

#### Individuals

Key individuals involved in service delivery in the implementation phase were the PRIME case managers and medical officers.

The case managers had an interest and willingness in mental health service delivery as they were employed by PRIME and were directly accountable to the PRIME team. They were trained for 9 days on HAP, CAP, the counselling relationship and psychoeducation (for psychosis). In addition to these training days, additional support was provided by the programme coordinator and the clinical psychologist. They conducted weekly supervision, 2 days quarterly refresher-training sessions and facility-based supervision. Skills acquired through facility supervision helped case managers to improve their overall competence to deliver psychological interventions.

Despite training of medical officers on mhGAP, they demonstrated very little motivation and a passive attitude towards mental health service delivery.

‘For the supervision of medical officer, we used different strategies, but I think out of those strategies none was successful. We organised a review session cum refresher training in the evening in Bhopal, but even then also medical officers attendance was not satisfactory. Then second strategy which we applied was supervision after OPD [out-patient department] hours, but that also did not work.’ Programme director, PRIME, India

#### Care processes: engagement, execution/implementation and improvement

##### Engagement

The programme coordinator supervised the work of case managers, but in addition to this, played an important role as an internal implementation leader and strengthened communication between the district mental health programme psychiatrist, district health officials, CHC medical officers, case managers and members of the PRIME team. Facility supervision by the programme coordinator helped case managers to develop and maintain better communication and relationships with CHC staff members. This had a big impact on overall engagement with the facility staff as well as improving the motivation of case managers.

‘After almost two years of implementation, now when I enter the facility I feel like a member of the facility and facility staff treats us as we are part of their facility. Our case managers are now considered as facility staff.’ Programme coordinator, PRIME, India

‘Whenever we face any difficulty in managing any situation, we give a phone call to our supervisors (the programme coordinator and the clinical psychologist). They always talk to us. If they could not receive our phone call at the same time then they always call us back and resolve all our queries. They guide us, if we face difficulty handling any patient case. They help us take decisions and they also convey to psychologist and psychiatrists in case of severe case.’ Case manager, PRIME

‘Supervisors (the programme coordinator and the clinical psychologist) help us a lot in developing our small-small skills. Programme coordinator used to visit us in the facility to ask how we are doing and if facing any difficulty. And also, in the weekly supervision we improved a lot. We used to talk among us about our cases and the challenges we face in the CHC and used to discuss possible solutions to overcome those.’ Case manager, PRIME

##### Execution/implementation and improvement

The healthcare-platform-level processes were executed as per the process maps but there were major barriers related to feasibility and fidelity of intervention delivery. These barriers were identified with the help of process data collected using a digital information system on a daily, weekly and monthly basis. Various implementation strategies were designed to address these barriers and they were put into practice through regular supervision visits to CHCs. These are described below.

At the healthcare-platform level, barriers were faced in the detection, diagnosis, delivery of HAP and CAP, provision of pharmacological treatment and follow-up of patients.

##### Detection and diagnosis of depression and AUD

The process data indicates that 14 110 (11.5%) people were screened on the master chart out of 122 337 out-patient department attendees in these three CHCs. In total, 7.32% (*n* = 1033), 4.08% (*n* = 575) and 1.01% (*n* = 143) were enrolled in the programme for depression, AUD and psychosis, respectively. The low detection rate for depression and AUD at the facility level was a major challenge. This was because the case managers had to perform multiple service delivery processes, for example, taking case histories, and delivering HAP and CAP to new patients and follow-up patients in limited out-patient clinic hours. In addition to this, the number of patients enrolled in the HAP and CAP was quite high for two case managers in each of the CHCs to handle. As a result, further efforts to improve detection were not undertaken.

Medical officers did not record the diagnosis in the clinical notes (White Card), during the initial months of implementation phase. In order to address this barrier, case managers accompanied the patient during consultation with the medical officer, shared the score on the PHQ-9/AUDIT screening and also provided a brief presentation of symptoms. The clinical note-sheet was modified in a way that the medical officer only had to put a tick-mark against the names of the disorders and then write the medical prescription. This proved to be very helpful and in due course all patients screened by case managers started receiving the diagnosis from the medical officer.

##### Acceptability of psychological interventions

The acceptability of a psychological intervention as a ‘treatment’ by the patients was one of the most important barriers for the delivery of HAP and CAP.

‘In the beginning, patients wanted to get only medicines, they were not considering counselling as a part of treatment. Instead of anti-depressants, medical officers were prescribing multivitamin and b-complex to the patients with mild and moderate level depression. We explained patients that counselling is also a part of the treatment and it is not general talk.’ Case manager, PRIME

To improve the acceptability of psychological intervention among patients, the local language phrase such as ‘Boli ka ilaaz and Goli ka ilaaz’ (loosely translated as ‘treatment by pill, treatment by talk’) was used by case managers to explain to patients the difference between pharmacological and psychological treatments and the effectiveness of psychological treatments.

The HAP and CAP were culturally adapted for the Indian population and we used the Hindi translation of the manuals used in the PREMIUM trials.^12,15^ However, cultural aspects relating to gender, being from an agrarian/rural community, and inability to perceive ‘talk therapy’ as a treatment might have played an important role in low acceptability, which we were unable to explore in greater detail.

##### Feasibility of intervention delivery

The feasibility of delivering counselling sessions was another major barrier in the treatment process. A normal counselling session would last about 30–40 min, followed by lengthy documentation. Case managers found it very difficult to keep patients engaged and active throughout the session as the patients found it time consuming and showed unwillingness to sit throughout the session most of the time. This was addressed by sometimes delivering abridged sessions as mentioned in the PREMIUM trial protocol.

‘Sometimes it is very challenging when 3–4 patients come together. At any given time, we can address only one patient and other patients need to wait.’ Case manager, PRIME

Some patients living in the forest/tribal areas of the district used different dialects than the one spoken by case managers and this was another barrier encountered in the delivery of psychological interventions. The local terminologies were adapted for explaining symptoms of mental disorders, specifically symptoms of psychosis, in order to address the language barrier in explaining the symptoms from the master chart specifically to the tribal population and sometimes to the general population.

The challenges related to delivery of HAP and CAP were addressed with systematic facilitation through facility and weekly supervision, quarterly refresher training sessions and maintaining a facility register, which later improved case managers' competency in delivering psychological interventions. The facilitation was led by the programme coordinator (and the clinical psychologist) and included a review of performance indicators collected as part of the process evaluation, providing feedback on the same and addressing implementation barriers through consultation with the case managers.

##### Pharmacological management

Another important barrier in the implementation process was related to errors in psychotropic prescriptions. In order to address this the Plan-Do-Study-Act (PDSA) approach was adopted. First, the baseline data on prescription errors was collected, this was followed by a one-to-one meeting with the in-charge medical officers. Prescription error data were presented to them and ways to reduce prescription errors was discussed. A concise one-page document summarising the indication and dose of psychotropic drugs was given to them. Two such PDSA cycles were completed. At the end of 2 months, prescription errors reduced from 52% to 37%.

##### Patient follow-up

Low follow-up was by far the biggest barrier during the MHCP implementation. A total of 1033 patients with depression, 575 with AUD and 143 with psychosis were enrolled in the programme. HAP was offered to all patients with depression. The first session of HAP was completed by 1013 (98.06%) of participants. Only 100 participants (9.68%) completed four sessions of HAP. Fifty-six (5.42%) participants completed the HAP treatment. CAP was offered to all patients with AUD. The first session of CAP was completed by 557 (96.87%) of participants. Only 20 participants (3.48%) completed four sessions of CAP. 87 (15.13%) participants completed the CAP treatment. Out of 143 patients with psychosis, 141 (98.6%) patients/their caregivers received psychoeducation. A total of 45 (31.47%) patients visited for at least four follow-up visits for the consultation and treatment.

To motivate patients to come to follow-up session, case managers started sending telephone reminders 1 day prior to the scheduled follow-up sessions. Unfortunately, telephone reminders to patients did not lead to any improvement in follow-up rates (details in supplementary Appendix 3).

### Community platform

#### Interventions

The key evidence-based intervention at the community level was MHFA.^22^ MHFA is a single-session psychosocial intervention that includes listening non-judgmentally, assessing suicide risk, giving reassurance, encouraging the individual to use self-help strategies and to get appropriate professional help as appropriate, for example if they had suicidal plans.^22^

##### Source and relative advantage of evidence-based interventions

Case managers delivered the MHFA. They found this intervention very simple to understand and deliver and were not too concerned about the source of the intervention and in what context it was developed. As they did not have any prior training in other community-based mental health interventions/psychological first aid, it was difficult to assess whether the case managers found MHFA better/worse compared with other interventions.

##### Presentation of intervention

We did not undertake any adaptations to MHFA, but presented the content using mnemonics in Hindi. This helped the case managers to retain the content as well as deliver it to the individuals in the community.

#### Individuals

The key individuals involved in service delivery at the community-platform level were primarily case managers and community health workers such as ASHAs. A 2-day workshop was conducted for ASHAs to introduce them to presenting symptoms of priority mental disorders, about the referral pathways for these disorders and delivery of MHFA. However, there was little involvement of ASHAs in PRIME activities. ASHAs receive monetary incentives mainly from the maternal and child health programmes to provide community-based care. We could not meaningfully engage ASHAs as funds were not available either from PRIME or the government to give them incentives for community-based processes in the MHCP.

‘One reason of lack of involvement of ASHAs in the programme was that they had received only training, but they were not receiving any incentives if they identify any case in the community or screen them or refer them for treatment. Another reason was they remained engaged with other government programme activities too where they get incentives. So they prefer to do the work first from which they get incentives.’ Case manager, PRIME, India

The case managers had an interest and willingness in mental health service delivery as they were employed by PRIME and were directly accountable to the PRIME team.

#### Care processes: implementation and improvement

At the community-platform level, there were barriers related to awareness activities, detection of priority mental disorders and those related to home visits for patient follow-up and adherence support.

In the initial months of implementation, community awareness on mental health was generated through screening of an audio-visual film (Prakashdoot) and distributing awareness sheets in the villages. In order to screen the film ‘Prakashdoot’, a dedicated mobile van with a compact disc player and a screen was required. This van used to go to the villages during the day and screen the film. This activity required dedicated resources. After a few months of screening it was not feasible to allocate these resources, hence the screening was stopped.

Case managers had very limited time during a typical day to undertake activities at the community-platform level. They were able to visit and undertake community activities in only 136 villages (56.9% of total villages under three CHCs), which had a total population of 164 440. They visited these 136 villages at least once during the implementation period. They screened 4762 individuals (4.8% of the total adult population of approximately 98 664, assuming 60% of the total population is adult) and referred 1275 (26.8%) to the facilities for further management. All of them received MHFA at the community level. Among those who received referral slips, 274 (21.49%) individuals came to the CHC to seek services and 188 of them were enrolled in the PRIME programme and received PRIME interventions.

In addition to the above activities, case managers also planned to visit the homes of patients enrolled in the treatment programme. The programme coordinator developed village route maps to possibly cover more than one village in a day on the same route. Case managers maintained a follow-up diary to schedule follow-up visits in advance and made telephone contactl to patients 1 day prior to the home visit. A total of 519 home visits were undertaken by case managers.

### Role of context

Health systems/contextual factors play an overarching critical role in programme implementation both at the healthcare- and community-platform level. As a result, we have described them together in the section below.

#### Outer setting

##### External policies and stakeholder engagement

The Directorate of Health Services and the State Mental Health Authority play a very important role in planning and delivery of mental health services in the State. Engagement with these key stakeholders played a significant role in creating an enabling environment for the overall implementation of the MHCP. The Director of Health Services was involved in the work of PRIME since the development of the proposal. Due to his strong support, a memorandum of understanding between the PRIME team and the Department of Health Services was signed. PRIME implementation would not have been possible without this memorandum of understanding.

Interestingly, we found that the presence of a memorandum of understanding at State level was not enough to initiate programme implementation at the district level and in the three CHCs. There were significant barriers in the ‘inner settings’ domain that are described below. Support from the Directorate Of Health Services played an important role in addressing these barriers. The most important implementation strategy to address these barriers was to get a ‘directive’ passed from the principal secretary of the Department of Health Services.

Four key items were included in this document: (a) dedicated space for mental health services; (b) dedicated staff for delivery of mental health services; (c) ensuring availability of drugs and reporting on key performance indicators; (d) appointing one medical officer as a nodal officer in the CHC for coordinating the mental health programme.

‘Initially we faced difficulties in getting space for Mann-Kaksha, but the directive from the Principal Secretary helped us to get a letter from the Chief Medical and Health Officer (Sehore district) and then we were able to convince the CHC in-charge to allocate space for Mann-Kaksha.’ Programme director, PRIME, India

On the other hand, the Secretary of the State Mental Health Authority, initially had a very negative opinion about PRIME. He sent a letter to the Department of Health Services demanding the rationale for involvement of a foreign funded programme (PRIME) in improving mental health services in the State. In the first year of the programme, meetings were organised with the Secretary of the State Mental Health Authority and these were attended by the Director of Health Services and the research director of the PRIME consortium. The PRIME India Advisory Group was constituted and the Secretary of the State Mental Health Authority was invited to be the chair of this group. He generously accepted this offer and later made significant contributions in programme design as well as supporting programme activities.

At the community level, a community advisory board was formed to engage community representatives and leaders. The overall objective was to take their advice about various PRIME activities, especially community processes to improve acceptability of PRIME interventions. The role of the community advisory board members as external change agents had a major impact on programme implementation.

#### Inner setting

There were several health-facility/CHC-level factors that proved to be major barriers in the process of implementation. These barriers related to the communication between various service providers, organisational culture, implementation climate and access to information.

##### Structural characteristics and communication

There were six medical officers in Shyampur CHC, seven in Bilkisgunj CHC whereas there were only two medical officers in Doraha CHC. One medical officer from each CHC was appointed as nodal officer for mental health services. Although we had trained all the medical officers in these CHCs, only one medical officer from each CHC (the nodal officer) was actively involved in providing mental health services. During the initial months, there was very little communication between the CHC medical officers and the PRIME case managers and programme coordinator.

Case managers made active efforts to establish communication with CHC medical officers and other staff members such as nurses and pharmacists, for example, helping CHC teams in their health programmes such as the polio campaigns.

##### Implementation climate

CHCs initially were not too keen on providing space and even asked the PRIME programme coordinator to set up Mann-Kaksha outside the CHC facilities. They were not ready to report on indicators, drug procurement was not there and there was no priority for mental disorders. Sustained efforts by the programme coordinator at the district administration level and support from the case managers at the facility level helped to first set up the drug procurement system and the information system followed by setting up of the Mann-Kaksha in each of the facilities. This helped to improve the overall implementation climate at the health-facility level.

##### Readiness for implementation

In Madhya Pradesh, medical officers in the government service run their own clinics within the premises of CHCs. This results in limited availability of medical officers in the CHC and effectively they provide services only for 2–3 h during the day. There are issues related to doctor–patient communication and relationships as well. Doctors in India in primary care provide 2 min on average to patients.^23^ This severely affects the way patients, especially with mental disorders are assessed.

‘Actual OPD [out-patient department] hours are from 8:00 am to 1:00 pm but patient rush is high between 10:30 to 11:00 am because doctors come at this time usually.’ Programme director, PRIME, India

Detection could have improved if medical officers and other CHC staff (for example nurses) actively assessed or screened and referred potential patients to the case managers. However, medical officers referred very few patients on their own to case managers for screening.

##### Access to information

Medical officers found it very difficult to access the mhGAP Intervention Guide during their busy clinics. To address this barrier, information in the mhGAP Intervention Guide pertaining to the symptoms and management of three priority disorders was presented in the form of a chart that was placed on the working desk of the medical officers. A WhatsApp group for the case managers and the PRIME team members was created. If the case managers faced any administrative problems related to the implementation or had any query regarding intervention delivery, they contacted the programme coordinator/clinical psychologist, which helped in quick resolution of the problem.

## Discussion

**Fig. 2:**
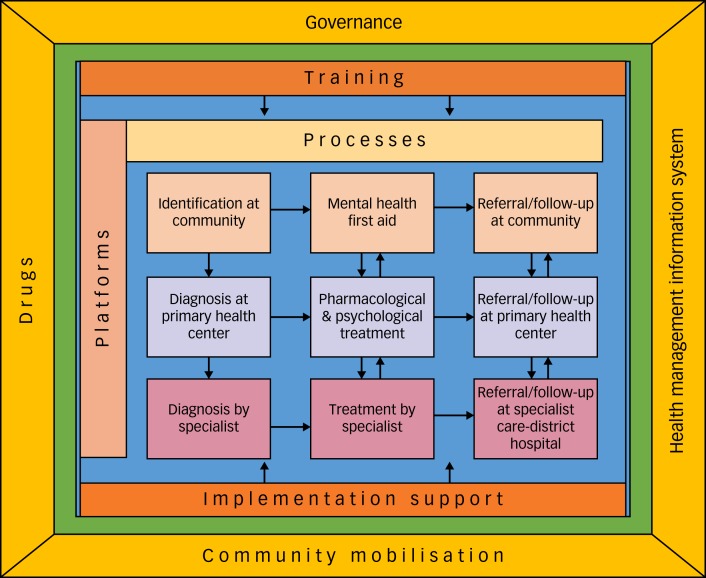
PRogramme for Improving Mental health carE (PRIME) India implementation model.

Implementation of the MHCP in Sehore district in Madhya Pradesh, India, has demonstrated that it is feasible to establish structures (for example Mann-Kaksha) and operationalise various processes to integrate mental health services in primary care. The key lessons from the mixed-methods evaluation of the PRIME MHCP in Sehore district can be summarised as: (a) clear ‘process maps’ of clinical interventions as well as implementation steps are very helpful in monitoring/tracking the progress; (b) in addition to the training of service providers, implementation support from an external team is essential to provide clinical supervision and address the implementation barriers; (c) enabling packages of the MHCP play a crucial role in strengthening the health systems and improving the context/settings for implementation; and (d) engagement with key community stakeholders and incentives for community health workers (ASHAs in the case of India) are necessary to deliver services at the community-platform level. The lessons learned are summarised in the PRIME India implementation model (Fig. 2).

In order to achieve the desired health and social outcomes it is important to have effective interventions, effective implementation and enabling contexts.^24^ The starting point for the MHCP was evidence-based interventions such as the mhGAP Intervention Guide, and HAP and CAP, which were shown to be effective in controlled settings. MHCP implementation was an attempt to understand how to integrate these evidence-based interventions in a low-resourced primary care setting. Establishment of a dedicated room for provision of mental health services (Mann-Kaksha), appointment of a case manager to oversee care processes and provision of clinical supervision and implementation support by an internal implementation leader (PRIME programme coordinator) were the critical factors, without which implementation of the MHCP would not have taken place.

There were major barriers related to the feasibility and fidelity of intervention delivery. Very low dosage of psychological interventions and errors related to psychotropic prescriptions remained as significant challenges until the end. Two case managers per CHC also faced time constraints and it was not feasible for them to increase the screening of individuals in CHCs and in the community beyond a certain limit (approximately 10% of the out-patient clinic attendants and 5% of the community members were screened during the 2 years), which resulted in a limited impact on the facility- and community-level outcomes. Our findings suggest that there was minimal involvement of the CHC medical officers and the community health workers in the overall implementation process. It may have been almost nil (as observed during the pilot implementation) in the absence of enabling packages in the MHCP. Various activities undertaken in collaboration with the senior officials in the government to streamline the programme governance, drug procurement and supply chain management, operationalisation of information systems and regular meetings helped to improve the participation of CHC medical officers. However, improving the ‘implementation climate’ at the facility level and the ‘readiness to change’ of the existing service providers in the government system, still remains a major challenge to be addressed.

A major challenge in global health is the lack of the impact of a multitude of global, regional and national plans, initiatives, policies and interventions, on the health outcomes. The translation gap between ‘evidence’ and ‘practice’ is well recognised in both high as well as and low- and middle-income countries (LMICs).^25,26^ A review of healthcare delivered to adults from the USA shows that up to 45% of patients fail to receive treatments that have been shown to be effective, and 11% receive treatments that are not needed or potentially harmful.^27^ In LMICs, diagnoses are frequently incorrect for serious conditions, such as pneumonia, myocardial infarction and newborn asphyxia.^28^ Mothers and children receive less than half of recommended clinical actions in a typical preventive or curative visit, less than half of suspected cases of tuberculosis are correctly managed, and fewer than one in ten people diagnosed with major depressive disorder receive minimally adequate treatment.^28^ Globally, the quality of care delivered in the health systems remains a major concern.^28^ There are several health systems and implementation-related challenges to integrate mental health in primary care. These have been identified in diverse settings such as Kenya,^29^ Brazil,^30^ Jordan^31^ and Lebanon^32^ as well as in other PRIME settings.^33^ The findings of our study should be seen in this broader context.

One of the major limitations of the MHCP evaluation was our inability to measure implementation effectiveness in clear quantitative terms (for example by using validated scales such as stages of implementation completion^34^) and also the failure to measure the baseline readiness to change (for example by using validated scales such as organisational readiness for implementing change^35^) and its impact on MHCP implementation. Another important limitation was the use of CFIR at the analysis stage only. In hindsight, we feel that if the CFIR was used to design the study then it would have been possible to obtain richer information related to implementation barriers. Patients were not directly involved in evaluation of MHCP implementation and this should also be considered as one of the limitations of this study. However, a separate qualitative study was conducted with patients to understand the impact of MHCP implementation as well as to further explore the barriers in service utilisation. The findings of this study will be published later. Despite the major shortcomings related to effectiveness of implementation and creating an enabling environment at the CHC level, the key lessons learned from PRIME implementation in Sehore district led to major system-level changes at the State level.

### Policy and practice implications

Based on the lessons learned and experiences of PRIME implementation in Sehore district (mentioned above), the Government of Madhya Pradesh decided to launch the SOHAM (Scaling up Opportunities for Healthy and Active Minds) initiative to scale-up mental health programmes in all 51 districts in the State covering a population of 75 million.^36^ The integrated model of care is based on the MHCP designed by the PRIME project in Sehore district. This scaled-up mental health programme is now led by the Department of Health Services and is included under the National Health Mission, which oversees implementation of all public health programmes in the State. Until 2014, mental health programmes were led by the Department of Medical Education whereas all other health programmes were under the Department of Health Services. This led to fragmentation of services and non-integration with the public health system in the State. This was identified as one of the major challenges in the situational analysis.^5^ Implementation of the PRIME MHCP in close collaboration with the Department of Health Services and the District Mental Health Programme during 2013–2016 along with several discussions with the senior-level decision-makers in the Department of Health Services might have resulted in this major shift. There was no allocation of funds by the State government for mental health programmes until 2011 and even the funds allocated by the Central government were underutilised. With the scale-up of mental health programmes across the State, the situation has completely changed. The State government allocated a dedicated budget in the financial year 2017–2018 as part of its general health budget to support State-wide scale-up of mental health services. Financial integration of SOHAM in the general healthcare budget is a very affirmative step and a key signal indicating the government's commitment to scaling-up of mental health services in the State. A senior-level official of the rank of the deputy Director of Health Services was appointed in 2015 to oversee implementation of mental health services across the State. The Department of Health Services has now established ‘Mann-Kaksha’ in all 51 district hospitals in the State and a minimum of two nurses and one medical officer from each district are trained to provide mental health services. The suggestion to appoint case managers in district hospitals was not accepted, but it was decided that nurses will fulfil the functions of case managers. Operationalisation of ‘Mann-Kaksha’ to strengthen mental health services was awarded as a best practice by the National Health Mission.^37^ The work initiated by the PRIME will now be continued in some form by the Government of Madhya Pradesh. However, it is critical to address the following barriers related to service delivery: low detection of mental disorders, errors in pharmacological prescriptions, low follow-up and completion rates of psychological interventions, additional burden and lack of motivation of healthcare providers.

### Implications for future research

Currently the government is providing training to mental health teams in the district, but is struggling to address structural, contextual and attitudinal barriers to motivate and support the healthcare team to implement evidence-based interventions in a collaborative manner with the ultimate goal of achieving optimal patient outcomes and implementation fidelity. Given the limited resources it is critical to find ways in which training and implementation support can be provided. Building on the experiences of MHCP implementation, a new National Institute for Mental Health funded grant entitled ESSENCE (Enabling translation of Science to Service to ENhance Depression CarE) will try to assess the comparative effectiveness of technology-enabled interventions to train non-specialist health workers and provide them with implementation support (through a digital platform) to address the key barriers to the coordinated delivery of evidence-based clinical treatments for depression at the primary healthcare level.

In conclusion, PRIME MHCP implementation provides a good starting point to establish the collaborative care model of integrated mental health service delivery in low-resource primary care settings. The lessons learned from the PRIME implementation model could be potentially used to scale-up mental health services across India.

## Supplementary material

For supplementary material accompanying this paper visit http://dx.doi.org/10.1192/bjo.2019.53. click here to view supplementary material
