# Supplementary material for: Delivering a complex mental health intervention in low-resource settings: lessons from the implementation of the PRIME mental healthcare plan in primary care in Sehore district, Madhya Pradesh, India
Source: BJPsych Open. 2019 Jul 29;5(5):e63. doi: 10.1192/bjo.2019.53 (PMC6669881; doi:10.1192/bjo.2019.53)
Supplement: Supplementary file 1 [file S205647241900053Xsup001.zip › BJO1900053_supplementary material.DOCX]

**Delivering a complex mental health intervention in low resource setting: Lessons from the implementation of PRIME Mental Health Care Plan in primary care in Sehore district, Madhya Pradesh, India.**

**Supplementary Fig. 1 Process Maps**

Supplementary Appendix 1

Summary of the barriers and facilitators in MHCP implementation

| **CFIR Domain** | **MHCP Package** | **Key description** | **Barriers/Facilitators** | **Key actions to address the barriers** |
| --- | --- | --- | --- | --- |
| **Healthcare Platform** | | | | |
| Interventions | Pharmacological Interventions | WHO’s mhGAP intervention guide (for three priority disorders) | **Facilitators:**   1. Highly credible source |  |
|  | Psychosocial Interventions | Healthy Activity Program (HAP) for Depression Counselling for Alcohol Problems (CAP) Psychoeducation for Psychosis | **Barriers:**   1. No patient information booklet 2. Interventions were evaluated in controlled setting, no previous learnings of intervention delivery in real settings | 1. No major adaptation in core components of HAP and CAP. Translated into Hindi. 2. Additional materials were developed in Hindi (Smile Card) for patient information |
| Individuals | Governance/Human Resource | Key individuals involved for service delivery were Case Managers and medical officers Key individuals involved for support processes were PRIME Programme Coordinator and Clinical Psychologist | **Facilitators**:   1. Case Managers had interest and willingness as they were directly accountable to the PRIME team 2. PRIME Programme Coordinator and Clinical Psychologist were part of the PRIME Implementation Support Team   **Barriers:**   1. Medical Officers had little motivation and a passive attitude as they were not directly accountable for mental healthcare services | 1. Training and supportive supervision improved motivation of Case Managers 2. Continuous liaising and Quality Improvement work helped to improve medical officers’ engagement with the programme |
|  | Capacity Building | Medical Officers were trained in mhGAP base training Case Managers received 9 days training in delivery of HAP, CAP and Psychoeducation for psychosis patients/caregivers. Quarterly refresher trainings were also conducted. Weekly supervision and facility-based supervision was done for case managers. | **Facilitators**:   1. Active participation enhanced Case Manager’s knowledge and skills 2. Medical Officers routine work was not disturbed as the mhGAP training was conducted immediately after OPD hours   **Barriers:**   1. Medical Officers perceived additional burden in providing mental health services 2. Medical Officers were unwilling to participate in supervision process | 1. PRIME Implementation Support team provided regular clinical supervision to Case Managers and helped in addressing several implementation barriers |
| Care Processes | Governance | MHCP and TOC map provided the overall framework to plan various implementation tasks | **Facilitators:**   1. Process map served as the basic guideline for the execution of service delivery 2. Process data helped in identifying the barriers | 1. Process maps were reviewed after six months of implementation 2. PRIME Implementation Support team supported the case managers in implementation of various processes as per the process map. |
|  | Detection/ Diagnosis | Case Managers screened the patients attending out-patient clinics using mhGAP master chart and PHQ-9 (for depression) and AUDIT (for AUD) Case Managers shared the result of screening with medical officers who made the diagnosis | **Facilitators:**   1. Presence of the Programme Coordinator and the Clinical Psychologist helped to identify barriers and in the supervision sessions solutions were designed with the Case Managers to address these barriers.   **Barriers**   1. Low detection rate at facility 2. Medical Officers did not diagnose and refer the cases on their own 3. Medical Officers were not writing the diagnosis on clinical note-sheet (White Card) | 1. Clinical note-sheet was modified. Medical Officer only had to put a tick-mark against the names of the disorders and then write the medical prescription |
|  | Treatment | Pharmacological treatment as per mhGAP guidelines Delivery of HAP/CAP and Psychoeducation as per protocol Specialist clinics conducted for psychosis patients | **Facilitators:**   1. Availability of essential psychotropic drugs in CHCs. 2. Availability of translated version of HAP and CAP for delivery of psychological interventions.   **Barriers**   1. Low detection rate at facility 2. Medical Officers did not diagnose and refer the cases on their own 3. Medical Officers were not writing the diagnosis on clinical note-sheet (White Card) | 1. Used phrases such as ‘treatment by pill, treatment by talk’ to improve acceptability of psychosocial interventions 2. Local terminologies were adapted for explanation of various terms 3. Plan-Do-Study-Act (PDSA) approach was adopted to reduce prescription errors |
|  | Referral/Follow-up | Referral/Follow-up as per treatment protocol | **Facilitators:**   1. Record of patient visits in the master register helped the Case Managers to schedule reminder calls for patients.   **Barriers:**   1. Low follow-up rate | 1. Telephonic reminder one day prior to schedule and maintaining follow-up diary |
| **Community Platform** | | | | |
| Interventions | Psychosocial Intervention | Mental Health First Aid (MHFA) | **Facilitators:** Easy to deliver in the community setting |  |
| Individuals | Governance/Human Resource | Key individuals involved for service delivery were Case Managers and Community Health Workers (Accredited Social Health Activists (ASHA)) Key individuals involved for support processes were PRIME Programme Coordinator and Clinical Psychologist | **Facilitator:**   1. Case Managers had interest and willingness as they were directly accountable to the PRIME team 2. PRIME Programme Coordinator and Clinical Psychologist were part of the PRIME Implementation Support Team   **Barrier:**   1. Limited involvement of Accredited Social Health Activists (ASHA) due to lack of financial incentives and low priority to mental health in their routine work | 1. Multiple discussions held with MoH, however the issue of incentives could not be addressed as there was no provision for incentives |
|  | Capacity Building | Case Managers and Community Health Workers received two days’ orientation training on mental health which included delivery of Mental Health First Aid | **Facilitators**:   1. Active participation enhanced Case Manager’s knowledge and skills   **Barriers:**   1. In absence of financial incentives, it was difficult to ensure involvement of ASHA workers. As a result, it was difficult to assess if the training resulted in any change in practice at the level of ASHAs. | 1. PRIME Implementation Support team provided regular supervision to Case Managers and helped in addressing several implementation barriers |
| Care Processes | Governance | MHCP and TOC map provided the overall framework to plan various implementation tasks | **Facilitators:**   1. Process map served as the basic guideline for the execution of service delivery 2. Process data helped in identifying the barriers   **Barriers:**   1. Community level processes were suboptimally implemented due lack of human resources | 1. Process maps were reviewed after six months of implementation 2. PRIME Implementation Support team supported the case managers in implementation of various processes as per process map 3. Community level processes were modified to adjust and improve service delivery within the available resources |
|  | Identification | Identification of the cases using mhGAP master chart | **Facilitators:**   1. Programme Coordinator worked with the Case Managers to plan activities on community platform. 2. Availability of an evidence-based psychological first aid intervention (Mental Health First Aid).   **Barriers:**   1. Case Managers had very limited time during a typical day to undertake activities on community platform 2. Involvement of community health workers was remaining limited mainly because they were not receiving financial incentives either from PRIME or DHS | 1. Route map was developed to cover more villages of same route. 2. Follow-up diary was maintained and telephonic calls were given to the patients one day prior to the home visit |
|  | Treatment | Delivery of Mental Health First Aid to cases identified positive on mhGAP master chart | 1. | 1. |
|  | Referral/Follow-up | Case Managers did home visits to follow-up with patients enrolled in the programme | 1. | 1. |
|  | Community Mobilisation | Screening of ‘Prakashdoot’ in implementation villages Distribution of mental health awareness sheets in the community | **Facilitators:**   1. Members of Community Advisory Board provided guidance and supported community level activities.   **Barrier:**   1. Lack of resources to continue screening of Prakashdoot |  |
| **Context/Health Systems related factors** | | | | |
| Outer Setting | Governance | Directorate of Health Services (DHS) and the State Mental Health Authority (SMHA) play important role in mental health programme implementation at the state level. | **Facilitators:**   1. Strong support from the Director of Health Services. 2. Memorandum of Understanding (MoU) was signed with DHS 3. ‘Directive’ passed by the DHS to provide support to establish Mann-Kaksha, procurement of drugs, dedicated human resource for implementation of mental health programme and reporting of key performance indicators.   **Barriers:**   1. Initially SMHA had a very negative opinion about PRIME work | 1. PRIME India Advisory Group (PIAG) was constituted and the Secretary of the SMHA was invited to be chair of PIAG. He accepted this offer and later supported PRIME activities. |
| Inner setting | Governance/HMIS/Drugs | Structural Characteristics and Communication | **Barriers:**   1. All medical officers of the facility could not be involved 2. Very little communication between the CHC Medical Officers and PRIME Case Managers and Programme Coordinator during initial months 3. The communication between the DMHP Psychiatrist and the medical officers was also very poor | 1. Strong facilitation by PRIME Programme Coordinator to improve communication 2. Case Managers made active efforts to establish communication within the CHC 3. Organisation of regular specialist clinics to improve the linkages between the DMHP psychiatrist and the CHC medical officers |
|  |  | Culture | **Barriers:**   1. Limited time availability of Medical Officers in the CHC as they are more engaged in private practice. |  |
|  |  | Implementation Climate | **Facilitators:**   1. Local drug procurement provision was available.   **Barriers:**   1. Initially space was not provided for Mann-kasha 2. No reporting on mental health indicators 3. No particular need to address mental disorders | 1. Facilitation meetings with Chief Medical Officer and facility in charge were conducted by the PRIME Programme Coordinator 2. The continued presence, work and engagement efforts by PRIME Case Managers. |
|  |  | Access to information | **Barrier:**   1. Difficulty was faced by the medical officers to access mhGAP intervention guide during their busy clinics. | 1. A chart containing information about the diagnosis and management of priority disorders was designed and placed under the working desk of the medical officers. |

**Supplementary Appendix 3**

**Telephone reminders**

Case Managers observed that many a times phones were switched off or someone else from the neighborhood picked the call and assured that they will inform the patient about follow-up visit. After the completion of implementation phase, participants enrolled in depression treatment cohort group, were contacted by Case Managers on their mobile phones. Out of 1033 participants, 50.1% couldn’t be contacted as they had not provided their mobile numbers to CMs. 26.5% participants reported that they had been feeling symptomatic relief, therefore, they discontinued the treatment; in 6.9%, either call was not picked or mobile number was wrong. Further, in 0.2% participants, death and/or migration were reported, and 3.1% participants reported that they didn’t find PRIME interventions effective, therefore stopped the treatment. In 2.9% participants, treatment for some other health issue(s) (not necessarily for mental health) and in 0.5% financial constraints resulted in inability to come to CHC to continue care. Similar to depression cohort, 41.9% of AUD participants did not register their mobile number with CMs. 31.0% reported experiencing symptomatic relief, as a result discontinued treatment. In 1.2% participants, migration/death was reported and in 4.4% either call was not picked or the phone number was wrong. 3.5% participants didn’t find the interventions effective and discontinued the treatment and in 0.7% financial constraints resulted in inability to come to CHC to continue care.
