# Supplementary material for: Delivering a complex mental health intervention in low-resource settings: lessons from the implementation of the PRIME mental healthcare plan in primary care in Sehore district, Madhya Pradesh, India
Source: BJPsych Open. 2019 Jul 29;5(5):e63. doi: 10.1192/bjo.2019.53 (PMC6669881; doi:10.1192/bjo.2019.53)
Supplement: Supplementary file 1 [file S205647241900053Xsup001.zip › BJPsych_Open_Appendix_Smile_Card_PSY.pdf]

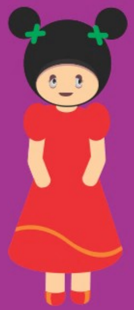

With Girl Child Our Future is Bright

PRIME | DMHP Sehore | PUBLIC HEALTH FOUNDATION OF INDIA | Department of Public Health and Family Welfare, MP | Sangath

DISTRICT MENTAL HEALTH PROGRAM, SEHORE

(District Mental Health Program, Sehore)

PID No. ____ / ____ / ____

PSY

Smile Card

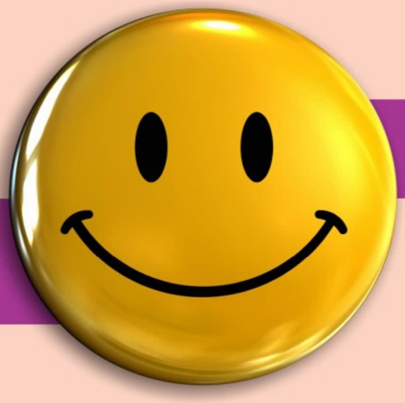

Name of Patient ....

Age .... Sex Male ☐ Female ☐

Fathers/Husband Name ....

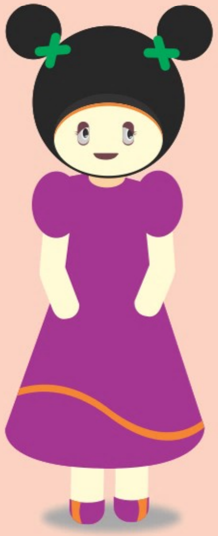

बेटी है तो कल है

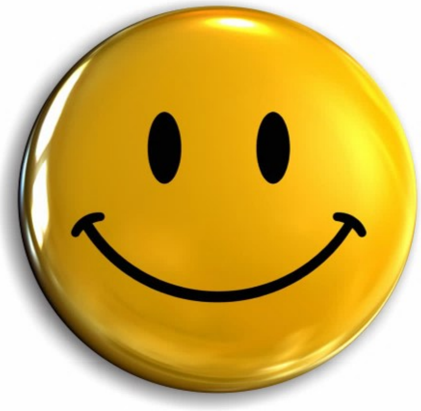

Presenting symptoms as per Master chart

Symptoms:

- Abnormal or disorganized behaviour (e.g. incoherent or irrelevant speech, unusual appearance, self-neglect, unkempt appearance) ☐
- Delusions (a false firmly held belief or suspicion) ☐
- Hallucinations (hearing voices or seeing things that are not there) ☐
- Neglecting usual responsibilities related to work, school, domestic or social activities ☐
- Manic symptoms (several days of being abnormally happy, too energetic, too talkative, very irritable, not sleeping, reckless behaviour) ☐

Symptoms And What You Can Do

Hallucinations - when your relative hears, or sees things that you do not hear or see. The person may talk to him or herself or otherwise seem to be responding to things that aren't there. Remember the experiences are real to the person having them and can be quite frightening and distressful. Hallucinations can involve any of the five senses.

Helpful

- Staying calm.
- Do distract the person if you can by: involving the person in something interesting; offering something to look at (e.g., newspaper article).
- Asking the person to help you find something (e.g., to find the newspaper); or to focus on some other activity.
- Do engage the person in pleasant conversation. You may ask about the experience if the person wants to talk about what they are seeing or hearing.
- Encourage the person to be with other positive and supportive people he or she knows well.

Not Helpful

- Blaming yourself or another family member.
- Panicking or getting angry.
- Don't try and figure out what he or she is talking about or to whom he or she is talking.
- Laughing about these hallucinations or strange talk.
- Don't ask him or her to try to force the voices to stop.
- Trying to minimize the experience for your relative. Remember it is real to them.

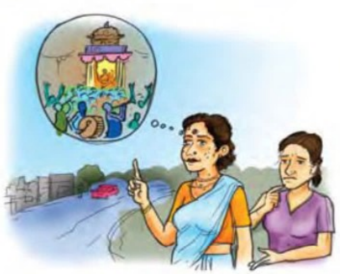

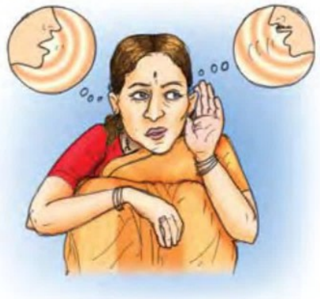

Sleeping or with drawing a lot of the Time or Sleeping at Odd Times

Helpful

- Do leave the person alone but make regular contact whenever he or she comes out.
- Letting your relative know you are there if needed.
- Remember that he or she may need sleep while recovering.
- Gently encourage other activities which are not too demanding
- (e.g., watching TV, washing dishes, pet care, etc).
- Do go out and enjoy yourself with other people.
- Do occasionally offer a cup of tea, coffee or juice.
- Ask what would be helpful.
- Do offer to help the person set up a schedule for sleeping and times to wake up.
- Involving the person in family activities if they are willing.

- **Not Helpful**
- Taking it personally or blaming yourself.
- Trying to coax the person out of his or her room.
- Worrying or fussing too much over your relative.
- Avoiding or isolating your relative.
- Inviting a lot of visitors home - it may be too overwhelming.
- Trying to force him or her to talk to people.

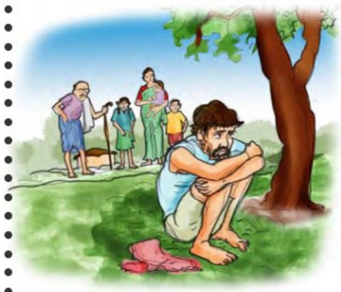

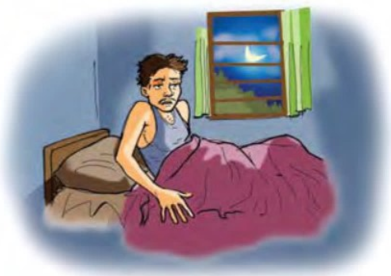

Inactivity and Not Feeling Like Doing Anything

Helpful

- If your relative says they are bored, offer or suggest some simple activities such as watching TV, listening to music, going for a walk, gardening, etc.
- Experiment with different activities to fi nd out what the person will enjoy. At first try activities that are passive (e.g., listening to or watching something).
- Try to have a regular daily routine so that things are predictable.
- Encourage him or her to join in or follow a daily routine.
- Encourage other members of the family to assist the relative. Everyone needs to help out.
- As your relative starts getting better, give simple daily chores to do. Break chores into small steps if they are difficult, as they are likely to be experiencing some difficulties with their memory. Give one instruction at a time.
- Try to make allowances for him or her if they need to do things like eating at unusual times (you can leave healthy snacks in the fridge).
- Offer incentives and praise for the times when your relative does the chores, even if the chores are not done perfectly. It's the effort which is more important than outcome.
- Remember that your relative may be destructible and may make mistakes or fi nd it hard to finish long jobs.
- With your relative's permission, do talk with your relative's, Medical officer or Psychiatrist about future plans. Get advice about when they are ready to do various things and how to encourage them to
- do these things.
- Remember to focus on the process rather than the outcome, as it will make everyone feel better.
- Find out about side effects of any medication as they can interfere

Not Helpful

- Don't insist on your relative doing too much or going out.
- Don't overwhelm him or her with too many suggestions at once.
- Your relative's thinking is usually slowed during recovery so short steps are more likely to be achieved.
- Suggesting activities or chores that are too complicated (e.g., a game of Scrabble or grocery shopping, and reading a technical book).
- Criticizing.
- Don't expect your relative to do things he or she is afraid of doing (e.g., going out to a party) or which he finds too confusing (e.g., writing letters).
- Do not argue with your relative.
- Don't give too many instructions at one time.
- Labeling your relative as 'lazy' - this label doesn't help either of you.
- Don't wear yourself out doing everything for your relative.

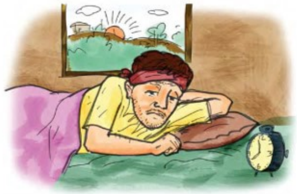

Aggressive Behaviour

- People with psychosis are often withdrawn. However, aggression may sometimes occur and you should know what to do if your relative becomes aggressive, so that you feel more able to cope in these situations.

Helpful

- Do develop a plan as to what to do in the event your relative's behaviour becomes diffi cult. Discuss with your Medical officer, supportive relatives and friends what role each of them will play in helping with the situation should it arise.
- Give clear direction such as "stop please".
- If he or she doesn't stop, leave the room or the house quickly.
- Do leave the person alone until they've calmed down. If you've left the house, a phone call may tell you if he or she is calmer.
- Do take any threats or warnings seriously and contact yourMedical officer or Psychiatrist, particularly if your relative has ideas of being persecuted and talks about "Getting them before they get me", etc.
- Afterwards you can say, "I know you were upset but we won't put up with aggressive behaviours or threats - EVER" or "You can tell us what you're angry about, but cannot threaten anybody".
- Discuss any threats and aggression openly in the family and with your Medical officer and Psychiatrist. Everyone needs to work together to help your relative manage these behaviours.
- Try to see what triggers the aggression and try to avoid the behaviour/situation (e.g., over crowding in
- the house, criticism, doing too much for the person, etc).
- If all else fails, it's OK to call emergency personnel (e.g. the police/ambulance) if you or your family needs help.

Not Helpful

- Saying angry, critical things, which may upset your relative.
- Arguing.
- Don't stay around if the person doesn't calm down.
- Ignoring verbal threats or warnings of aggression made to you or about others.
- Don't tolerate aggression to you or your family or others.
- Don't try to reason it out on your own – ask for help.
- Don't let yourself or the family become the only ones your relative depends on - this can create resentment, anger and isolation.

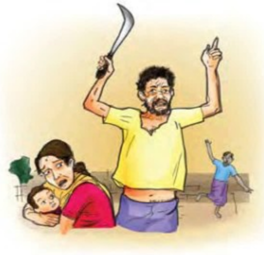

Strange Talks or Belief

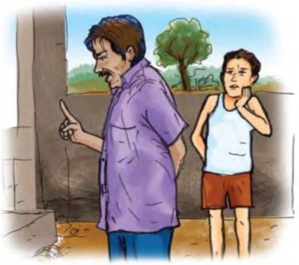

Helpful

- Gently and matter-of-factly identify that the strange ideas are not common or real.
- Show some understanding of the person's feelings (e.g., fear of the voices). Do encourage the person to talk openly.
- Do change the subject to something routine, simple, or pleasant in real life (e.g., what you're making for dinner).
- Do say when you think something is not real (e.g., 'the voices'), while acknowledging that they seem real to your relative.
- Help your relative to tell the difference between reality and fantasy by saying you think "It's your brain chemistry which is making something appear- it's not really out there".
- It may be appropriate to help the relative check out the facts about their beliefs. (e.g. getting a brain scan to show there is no implant in their head).
- Tell your relative that if he or she feels they must talk about the strange ideas, to do this only with certain people who are not worried by these ideas (e.g., Medical officer or other mental health professionals).

Not Helpful

- To allow other family members to make jokes or criticize the person.
- Don't argue about the strange ideas - arguing never changes the ideas and only upsets both of you.
- Spending much time listening to talk that makes no sense to you.
- Pretending to agree with strange ideas or talk you can't understand.
- Don't keep looking at the person or nodding your head if they are speaking strangely.
- Trying to enter his or her world and follow everything said.
- Don't keep up a conversation that you feel is distressing, or annoying, or too confusing for you. It's OK to say, "I'll talk to you later".
- Looking upset or embarrassed by strange talk. It's better to say clearly that you don't like or understand the strange talk.

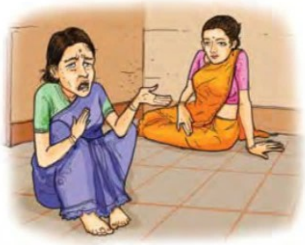

Fear of suicide

Helpful

- Listen to all your relative's feelings of depression but also point out that help is available.
- Do show appreciation of your relative's feelings and the fact that he or sheconfided in you.
- Contact your Medical officer or Psychiatrist if suicidal ideas persist.
- Discuss suicide and how to respond with your Medical officer. It's best to talk about your concerns and the available resources to support your relative and your family.
- Encourage your relative to be involved in pleasant, low key activities.
- Help the person to be with someone who accepts them so they don't feel isolated.
- It is important to let the person know you accept and care about him or her to reduce their sense of isolation.
- Consider whether any stressors can be removed which might be depressing your relative (e.g., too much pressure to go back to work, too many classes/courses at school, etc).

Not Helpful

- Don't panic if your relative talks about suicide, but do take his or her feelings seriously.
- Telling the person things like, "Pull yourself together".
- Not talking about this with a mental health professional.
- Keeping this a secret.

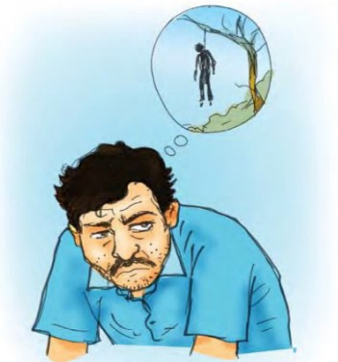

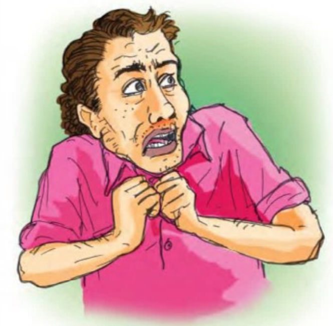

Odd or Embarrassing Behaviour

Helpful

- Do remember that you are not responsible for this behaviour.
- Ignore this behaviour if you can, especially if the behaviour is not serious.
- If you can't ignore the behaviour, ask the person clearly and pleasantly not to do that particular behaviour.
- If the person can't help the behaviour, ask him or her to do it only in their room.
- Do state clearly that the behaviour is not acceptable to others.
- If you can, change the environment so as to lessen the behavior (e.g., turn the TV off if it's upsetting).
- Find times to praise the person for acting more appropriately.
- If the behaviour seems to be set off by stress (e.g., too many visitors, being criticized, upsetting events, etc.) see if the stress can be reduced or lessened.

Not Helpful

- Telling yourself that the behaviouris a reflection on you or your family.
- Acting upset.
- Getting into long discussions.
- Letting other family members and friends pay attention to the behaviours or laugh at the behaviours.
- Nagging the person about the behaviour.

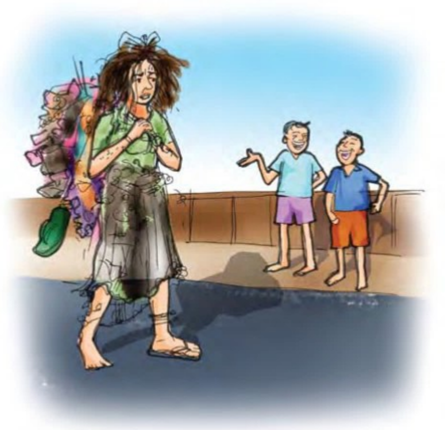

Alcohol or Street Drugs

Depending on the medication your relative is taking, the effects of drinking or taking other kinds of drugs (over the counter or street) can vary widely. Young people with mental disorders sometimes use marijuana to help with their symptoms such as sleeping difficulties or social anxiety. However, drugs such as marijuana can make symptoms worse and can trigger a relapse. If your relative takes these drugs or too much alcohol:

Helpful

- Remind him or her that the drugs are harmful.
- Do remind your relative about how alcohol may effect them.
- Assist your relative with developing ways (ideas) on how to say 'no' to offers of drugs or alcohol (e.g. I am on medication right now and I don't want to mix my drugs: I don't need drugs/alcohol
- to have a good time).
- Discuss how to cope with any stressors other than drinking or drug taking (e.g., fights with family, job pressures). Problem solving and developing plans for dealing with the stressors makes the
- relative feel more in control.
- Let your relative know that it's okay to let friends know that drugs/alcohol are not helpful when recovering from a chemical imbalance in the brain.
- Do discuss alternative ways of being social or being with friends without using drugs or alcohol. Assisting your relative to find other ways to enjoy themselves or to gain energy is really important.
- Your relative may need to learn new things (e.g., socializing with people who don't drink or take drugs, a new satisfying hobby, or doing volunteer work to gain a sense of achievement).

Not Taking Prescribed Medication

Helpful

- If the problem is a result of forgetting, gently remind the person when it is time to take the medicine.
- Use a dosette container to keep meds organized.
- Find a daily routine (e.g., breakfast, tooth brushing) when tablet taking can become a habit.
- Do remind him or her calmly that medication helps to keep/make him or her well.
- Do ask if he or she is having any side effects. Your relative may want to consider ways of helping with side effects: change of diet, water bottle, more exercise.
- Do talk to your Medical officer or Psychiatrist about your relative's difficulty with remembering to take medication and ask about the types of side effects, which may be expected.
- Informing your general practitioner about information on early psychosis, medication and your relative's side effects.
- If your relative refuses to take medication, let your Medical officerand Psychiatrist know if symptoms get worse or reappear.
- Calmly remind your relative that medication may be crucial to their recovery.

Not Helpful

- Nagging or threatening the person. This causes a loss of rapport and trust in you.
- Altering the prescribed dosage without psychiatrist's knowledge.
- Attributing every problem to not taking medication.
- Tricking the person by sneaking it into their food. They will notice the effects of medication and stop trusting you. This could put their health at risk if they decide to take the medication or are using street drugs.
- Supplementing the medication with herbs, vitamins or other medications without talking it over with the clinical team.
- Avoiding discussions about medication and side effects. Your relative may be on a number of medications and needs to be able to talk about them.

As Your Relative Recovers

- **Helpful**
- Slowly ask the person to get up earlier in the day and to do more things.
- Offer something to enjoy when he or she gets up, like a tempting breakfast or pleasant music.
- Do praise your relative for getting up, for being more social and for their effort.
- Help them with a plan for good personal hygiene, a healthy balanced diet and some physical activity.

Not Helpful

- Don't think you always have to be protective.
- Expecting your relative to stay ill or incapable of daily routine activities.

Note:

If your relative has been well for some time and develops sleeping difficulties or begins to withdraw again, discuss this with your relative. This may be a sign that your relative is relapsing.

Follow up

Time:........................	Date:........................
Time:........................	Date:........................
Time:........................	Date:........................

Follow up

Time:........................	Date:........................
Time:........................	Date:........................
Time:........................	Date:........................
