# Supplementary material for: Delivering a complex mental health intervention in low-resource settings: lessons from the implementation of the PRIME mental healthcare plan in primary care in Sehore district, Madhya Pradesh, India
Source: BJPsych Open. 2019 Jul 29;5(5):e63. doi: 10.1192/bjo.2019.53 (PMC6669881; doi:10.1192/bjo.2019.53)
Supplement: Supplementary file 1 [file S205647241900053Xsup001.zip › BJPsych_Open_Appendix_Smile_Card_Dep.pdf]

DISTRICT MENTAL HEALTH PROGRAM, SEHORE

(District Mental Health Program, Sehore)

PID No. ____/____/____

DEP

Smile Card

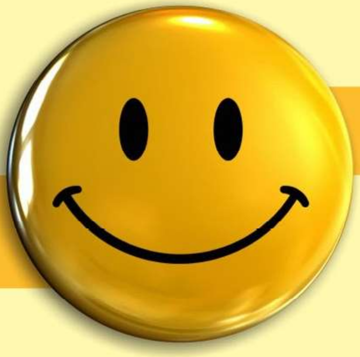

Name of Patient ....

Age .... Sex [ ] Male [ ] Female

Fathers/Husband Name ....

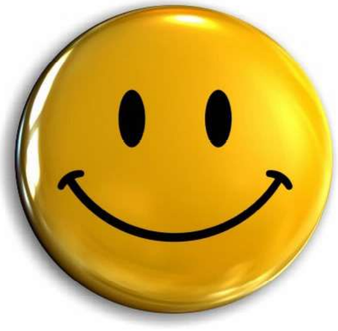

....

Presenting Symptoms as Per Master Chart

....

Symptoms:

- Low energy; fatigue; sleep or appetite problems ☐
- Persistent sad or anxious mood; irritability ☐
- Low interest or pleasure in activities that used to be interesting or enjoyable ☐
- Multiple symptoms with no clear physical cause (e.g. aches and pains, palpitations, numbness) ☐
- Difficulties in carrying out usual work, school, domestic or social activities ☐

How The Healthy Activity Program Will Help You

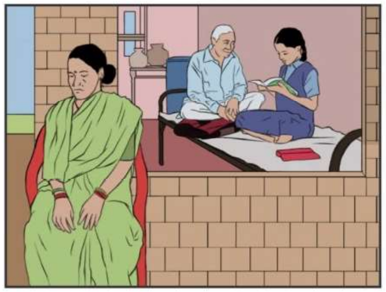

Stress in your life can make you feel sad and lose interest in activities. It can also lead to physical complaints like aches and pains, tiredness, disturbed sleep and appetite.

If these experiences last longer than a couple of weeks and begin to affect what you do each day and how well you are managing things in your life, then you may be suffering from a health problem that is related to stress. As a result, you may stop doing activities you used to enjoy and withdraw from family and friends. This can worsen your feelings of sadness and stress.

How Stress Affects Your Health

Please put a "✓" next to points that apply to you and your health

Stress In Our Lives	How This Affects Our Health	This Impact on Our Daily Life
☐ Family Problems	☐ Sleep & appetite problems	☐ Stop taking part in enjoyable activities
☐ Death of a loved one	☐ Tiredness & fatigue	☐ Withdrawing from people
☐ Unemployment	☐ Aches & pains	☐ Difficulty in keeping up your daily
☐ Work Stress	☐ Lack of interest in activities	
☐ Money Problems	☐ Sadness	
☐ Worry about the future	☐ Losing temper	
☐ Sickness in oneself or a family member	☐ Difficulty in concentrating	

Through our work together, you will learn how to do more enjoyable and useful activities. You will learn simple and effective ways of dealing with problems and tensions in your life. In this way, working with the counselor will help you to return to good health.

We will have around 6 sessions over the next 2 to 3 months. Each session will last between 30-40 minutes. We can arrange these sessions at your home, in the clinic or over the telephone, whichever is most convenient for you. The counseling provided in this clinic will not cost you anything.

It is very important to identify someone close to you who can support you in getting better. Please write the name of this person here:

Name ____

If you have any questions about the Healthy Activity Program, I will be very happy to answer them. I look forward to working with you.

Solving Life Problems

From time to time, all of us struggle to handle life problems. You might come across such problems yourself. Sometimes your inability to handle these problems can be due to your stress-related illness.

You could use the following 5 steps to try and tackle such problems effectively:

Define the problem clearly, be specific and try to narrow in on what exactly is the problem.

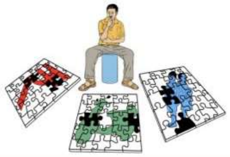

Try to find different solutions to the problem.

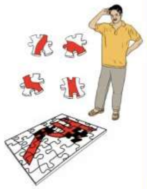

Think through each solution and check for advantages & disadvantages of the solution. choose the solution that promises the best result.

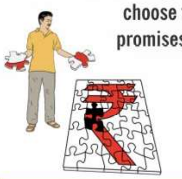

Plan to carry out the solution. Practice applying the solution.

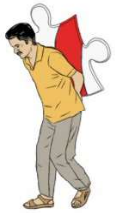

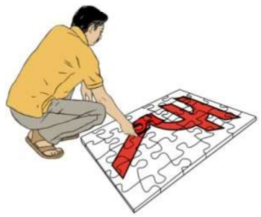

use a time limit to test the solution. Assess the outcome.

Solving Problems Worksheet

Use this page to ty out the steps for your problems:

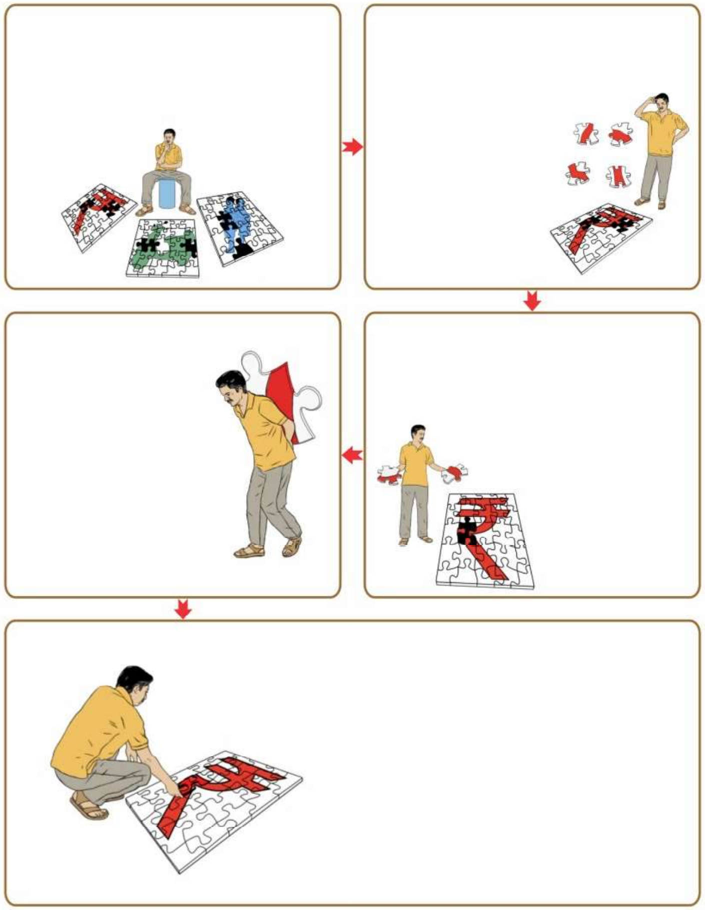

When You Have Problems With Someone Close To You

Below are ways to communicate when you have problems with someone close to you:

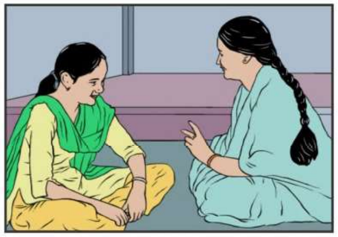

- Find a time to talk that is good for the other person.
- Focus on the current problem / argument (e.g. don't talk about all the mistakes the other person made in the past)
- Don't attack or criticize the other person. focus on the specific behaviors that are of concern to you (e.g. , saying "you hurt my feelings" rather than "you are a very unkind person")

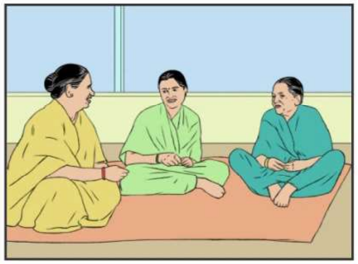

- Avoid using words such as "always" and "never". for example, instead of saying, " you never listen to me," you might say, " you interrupted me when we were talking this morning"
- Find someone to help you communicate better. this can be someone whom both of you trust and respect
- Be specific about what you want or don't want. For example, you might say. "I would like you to drive me to my appointment tomorrow" or "I don't want you to drink alcohol on the weekends"

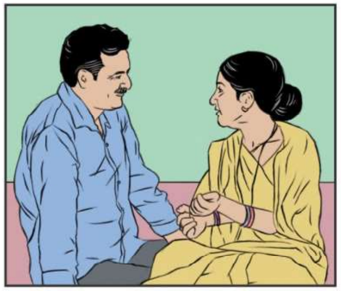

- Imagine yourself in the other person's position and acknowledge their experience ("I know you feel like I am not paying attention to you")
- Say how you feel as clearly as possible.Use "I" statements about how you feel and what you want. for example, say "I feel angry when you behave like this" rather than " you make me angry"

Dealing With Thinking Too Much

Do you think over and over about your problems or the cause of your problems?

Do you get caught in thinking negative thoughts, which worsens your mood and leads to more negative thoughts?

This often happens in people suffering from stress-related problems and if this is true for you too, here are some techniques you can try to control your thoughts:

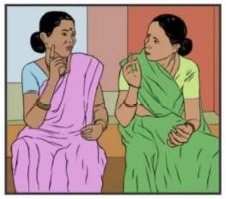

1. Work on solving the problem that is a cause of thinking too much

Identify the problem about which you are thinking too much and then follow the steps you have learnt to solve the problem. Remember that you can always ask for help with problems that are difficult to solve.

2. Pay attention to the sensations you feel in the present moment, rather than your thoughts

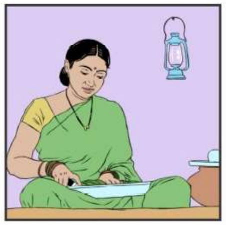

This is a way you can keep your mind focused in the present moment by directing your attention to what you feel, see, smell, hear, touch. You may find this difficult in the beginning, but with practice, it will become easier. Here are some examples of specific sensations on which you can focus:

- Notice all of the colours around you
- Notice the sounds that you hear around you, near and far
- Notice the smells that are present
- Notice the physical movements of your body, for example while you are cutting vegetables or washing dishes

3. Distract yourself from your thoughts instead of focusing on them

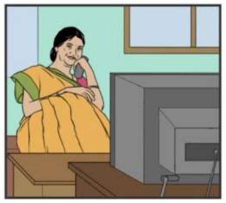

Distracting involves shifting your focus to notice something new or different around you. Some helpful activities to distract yourself may include:

- Physical activities (e.g. walking, household tasks, etc)
- Activities that you enjoy (e.g. watching a movie, talking to someone close to you)
- Activities that shift location (e.g. visit a neighbor, walking outside)

Breathing Technique To Relax

Why use the Breathing Technique:

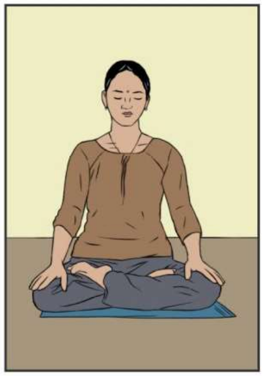

This Breathing Technique is used with people who report feeling anxious, tense, or who have difficulty relaxing, all of which are common among people who have stress related health problems

How to relax by doing the Breathing Technique

- Relax by lying or sitting in a comfortable position, preferably in a quiet room
- Close your eyes and after about 10 seconds, start concentrating on the rhythm of breathing. (You can do the same with open eyes if you are not comfortable closing your eyes)
- Now concentrate on taking slow, regular, steady breaths through the nose and, while breathing out, in your mind you can say "relax" or a similar word. If you are religious, you can use a word that has some importance to your faith, for example, "Om", "Praise the Lord", "Allah" or "Shanti"
- Continue the breathing for a few minutes
- If you experience your heart beating very quickly, uncomfortable feelings in your fingers or mouth, chest pain or any other physical discomfort while doing this breathing exercise, it may mean that you are breathing too fast; slow down the rhythm and start again

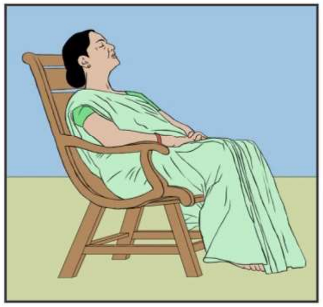

- Spend 5-10 minutes a day doing this breathing technique. If you practice daily, you will begin to feel the benefits of relaxation
- With experience, you may develop the skill to relax in a variety of situations, for example while sitting in the bus or office or cooking in the kitchen

How To Sleep Well

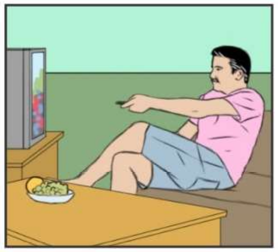

- Get up and go to bed at the same time every day even on weekends, regardless of the number of hours of sleep you got the previous night
- Allow yourself at least one hour to relax before bedtime.
- Avoid daytime naps. This will ensure you are tired at bedtime. If you just can't make it through the day without a nap, sleep less than one hour before 3 pm.

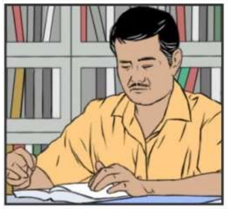

- Use your bed only for sleeping. Do not your fed for other activities such as reading or watching TV. Keep your bed only for sleeping.
- Avoid drinking tea or coffee after 5pm. Drinks with caffeine can make it difficult to fall asleep.
- If you are unable to fall asleep or fall back to sleep within 15-20 minutes get out of bed and find something else to do in another room.

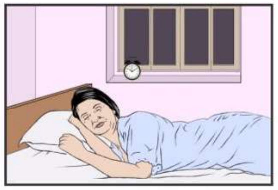

- do a calming activity that does not stimulate your mid very much.
- You also may try the breathing exercise which you have learnt.
- When you begin to feel drowsy, return to bed.
- Repeat this procedure as often as necessary during the night.

Activities to do and to not do when you awake during the night

Suggested Activities	Activities to Avoid
• Watching Television<br>• Listening to music<br>• Having a bath<br>• Drawing<br>• Reading	• During housework, cleaning<br>• Taking a walk or exercising<br>• Worrying<br>• Doing your office/job-related work<br>• Exposing yourself to bright light

Medicine for stress-Related Problems: What You need To Know

Your doctor may have prescribed medicine for your stress-related problem. The information on this page provides answers to some questions you may have about the medicine:

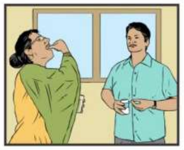

- **Are medicines useful for problems such as mine?**
- Medicine is sometimes useful for people with stress-related problems such as yours
- They will help in reducing your symptoms and improve overall health
- They can be given along with counseling treatment and the combine effect can be even better than just taking medicines alone

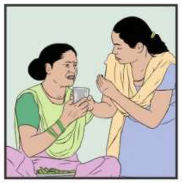

How long will it take for the medicine to work?

- It may take 2 to 4 weeks before you notice the full effect, but some patients respond earlier
- For best effect, it is important to take the medicine regularly as advised by the doctor

How long will I need to take me medicines for?

- You will need to take the medicine for 6 months or more to make sure you remain well
- You must talk to your doctor before you decide to take less or stop your medicine
- It is important to take the medicines as advised by your doctor, even if you are feeling well. If you stop the medicine too soon, your problems may return.

Will I have side effects if I take the medicine?

- Most patients have no side effects
- The medicine may sometimes cause minor side effects like headache, churning in the stomach, vomiting, sleepiness
- These are usually mild and settle down after 1 to 2 weeks of treatment
- Talk to your doctor if you are experiencing any side effects

Is the medicine addictive?

- The medicine is not addictive like some sleeping tablets or alcohol or cigarette smoking
- When you stop the medicine on the doctor's advice, you will not find yourself craving for the medicine

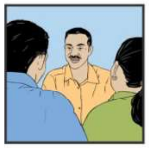

Contact The Doctor If:

- You are experiencing side effects
- You are not feeling better or you feel worse even after taking the medicine regularly for 4-6 weeks

Mood Ladder

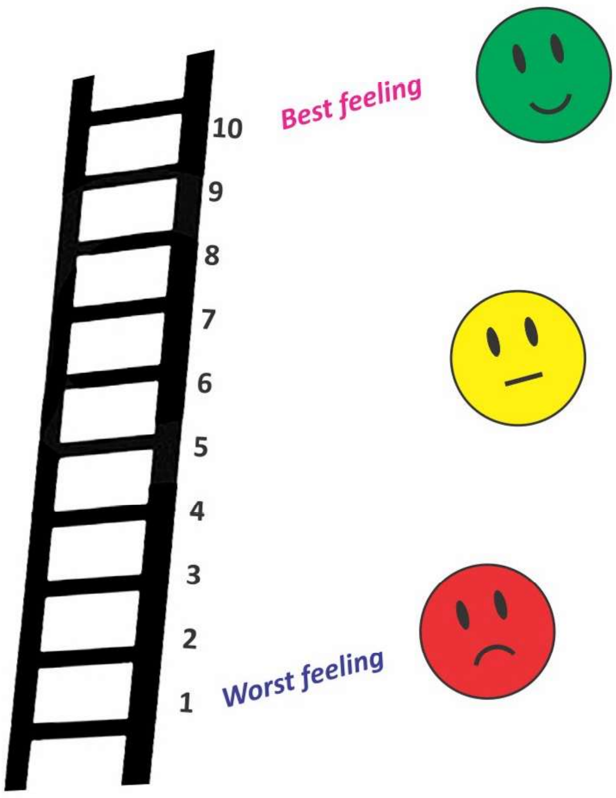

Activity Calendar

Date: ______________ Session No: ______________

How to fill this form:
For each part of the day, record your activities. Rate how you felt when you did each activity using a number between 1 and 10, with *"1 meaning the worst possible feeling"* and *"10 meaning the best possible feeling."*

Time of Day	Monday		Tuesday		Wednesday		Thursday		Friday		Saturday		Sunday	
	What did you do?	10 / 1	What did you do?	10 / 1	What did you do?	10 / 1	What did you do?	10 / 1	What did you do?	10 / 1	What did you do?	10 / 1	What did you do?	10 / 1
Morning														
Afternoon														
Evening / Night														

End of Treatment Form

Date:_____________

Complete this form with the counsellor to help you make a plan for remaining well after the completion of the Treatment.

- What are the Activities that make you feel good and that you need to continue to stay well?
  1.
  2.
  3.
- Which situations can increase your risk of developing health problems in the future?
  1.
  2.
  3.
- In these situations, what are the activities you may doing again which will make you feel worse?
  1.
  2.
  3.
- hat will help you to do the things that will make you feel better?
  1.
  2.
  3.

To remain healthy I will do following things

Date:_____________

I will stop doing following things	I will stop continue doing following things

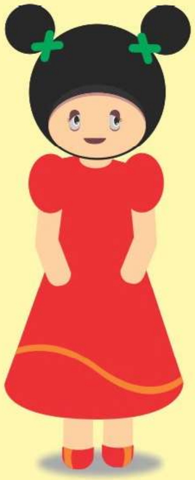

With Girl Child Our Future is Bright
