# Supplementary material for: Delivering a complex mental health intervention in low-resource settings: lessons from the implementation of the PRIME mental healthcare plan in primary care in Sehore district, Madhya Pradesh, India
Source: BJPsych Open. 2019 Jul 29;5(5):e63. doi: 10.1192/bjo.2019.53 (PMC6669881; doi:10.1192/bjo.2019.53)
Supplement: Supplementary file 1 [file S205647241900053Xsup001.zip › BJPsych_Open_Appendix_Smile_Card_AUD.pdf]

## APPOINTMENT CHART

| Serial No. | Date | Day | Session No. | Session Done X or ✔ | Case Manager Signature |
|---|---|---|---|---|---|
| | | | | | |
| | | | | | |
| | | | | | |
| | | | | | |
| | | | | | |
| | | | | | |
| | | | | | |
| | | | | | |
| | | | | | |
| | | | | | |
| | | | | | |
| | | | | | |

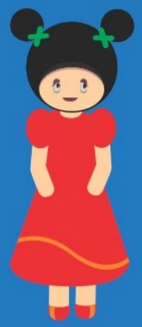

**With Girl Child Our Future is Bright**

PRIME | DMHP Sehore | phfi PUBLIC HEALTH FOUNDATION OF INDIA | Department of Public Health and Family Welfare, MP | Sangath

# DISTRICT MENTAL HEALTH PROGRAM, SEHORE

(District Mental Health Program, Sehore)

PID No. ____ / ____ / _ _ _ _

ALC

## *Smile Card*

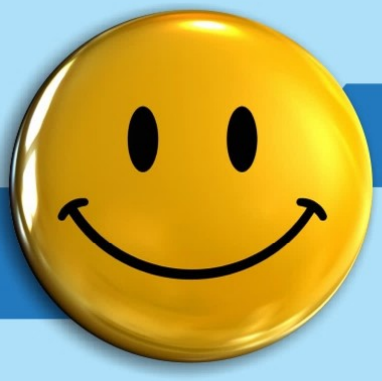

Name of Patient ............................................................

Age ............................................ Sex Male ☐ Female ☐

Fathers/Husband Name ............................................................

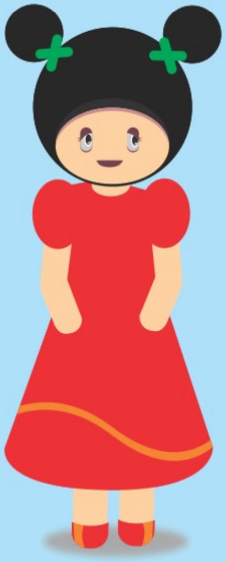

With Girl Child Our Future is Bright

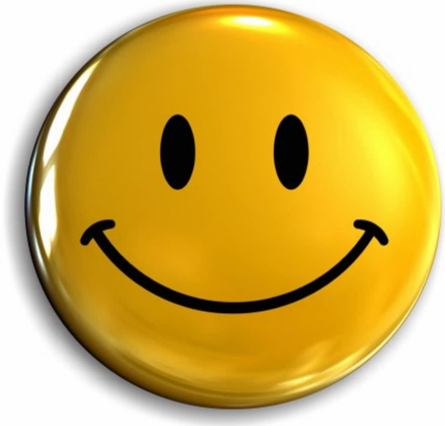

**Presenting symptoms as per Master chart**

**Symptoms:**

- Appearing to be under the influence of alcohol (e.g. smell of alcohol, looks intoxicated, hangover) ☐
- Presenting with an injury ☐
- Somatic symptoms associated with alcohol use (e.g. insomnia, fatigue, anorexia, nausea,vomiting, indigestion, diarrhoea, headaches) ☐
- Difficulties in carrying out usual work, school, domestic or social activities ☐

## Our Treatment For Drinking Problems?

- This treatment is to help people whose drinking is causing problems to their health or social life.
- It is a short counseling treatment of one to four sessions.
- Sessions will last upto an hour and will be on a weekly to fortnightly basis based on your convenience.
- The counseling treatment will help you: To understand the link between your drinking and any problems you may have. Learn how to change your drinking behavior. Learn how to make those changes long lasting.

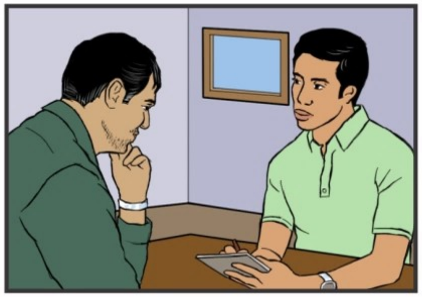

## Getting Help From Someone Close To You

- A Close Family Member Or Friend Can Help in many ways:
- Remind you to attend sessions.
- Accompany you to sessions for support / attend sessions with you.

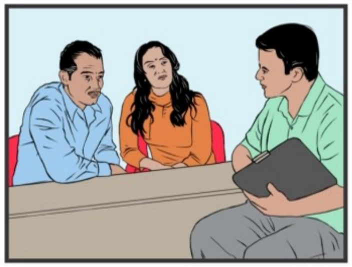

- Share any concerns he/she may have about your health with the counselor.
- They will also learn more about your problems and may think of ways in which they can contribute to your good health.
- We hope you will be able to bring along a dose family member or friend to your sessions, and we look forward to meeting her/him.



## Types Of Drinking Problems

- When a person's drinking has a negative effect on his life, he is considered to have a drinking problem.
- There are three categories of severity of drinking problems:
- Drinking which is likely to lead to physical, mental or social harm in the future.
- Drinking which has already started causing some physical, mental or social harm.
- In the most severe form, people feel that they need to drink, and their bodies crave alcohol. They often drink every day, and sometimes first thing in the morning. If they do not drink, they suffer from symptoms such as sweating, nausea, and in serious cases uncontrollable shaking, vomiting, or fits.

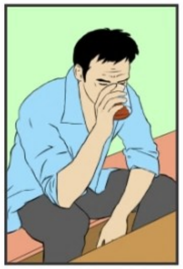

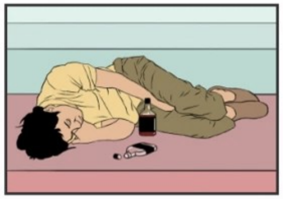

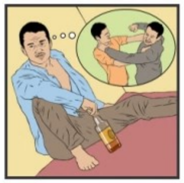

## Impact On Social & Family Life

- Family Problems:
- Family tension
- Quarreling and violence
- Missed family functions
- Family members may become anxious, depressed, and may drink excessively themselves
- Children may have behavior problems and perform poorly in school
- Financial problems and debts
- Social Difficulties:
- Conflict with colleagues Workplace accidents
- Frequent absence from work
- Repeated dismissals from work and long-term unemployment
- Arguments and fights
- Problems with the police
- Loss of friends

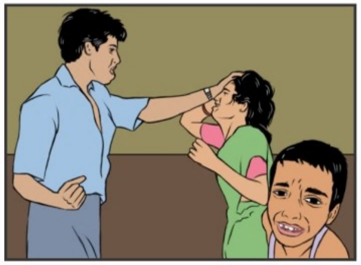

# Understanding Your Drinking

Date_______________

This summary gives you the results of your assessment and provides information about how alcohol affects you. This will help you create a clear picture of your drinking - information you will need to make choices about whether to change your drinking behavior.

## Where does your drinking fit in?

The average amount of alcohol you reported consuming per day was.
________________Your AUDIT score is___________which puts you in the category of:

(Tick ✓ as appropriate)

☐ **a) Harmful Drinking**
You drink at a level which causes harm to your physical or mental health or social/ family life.

☐ **b) Dependent Drinking**
You often feel that you need to drink, and your body craves alcohol. Because of this, you are likely to drink every day. You may sometimes drink first thing in the morning. If you do not drink, you may suffer from symptoms such as sweating, nausea or uncontrollable shaking.
Based on your typical drinking during one week, You spent from Rs.__________to Rs.__________ in the last month, on your drinking.

## Problems Related to Your Drinking

You reported that in the past year, your drinking had a harmful effect on:
(Tick ✓ as appropriate)

☐ Your friendships or social life

☐ Your physical health

☐ Your mental health

☐ Your home life or marriage

☐ Your work, studies, or employment opportunities

☐ Your financial position

☐ Any other (specify):______________________

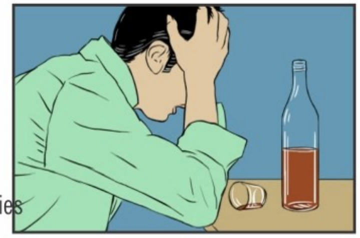



# Are you ready for change?

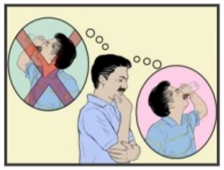

You have not thought about changing your drinking behavior. ☐

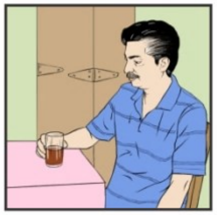

You have started thinking about making a positive change to your drinking behavior, but at the same time have a strong feeling for continuing drinking. ☐

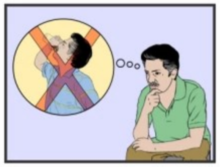

You have already decided to either control or completely stop your drinking. ☐

## Why It Is Important That You Take Charge Of Your Drinking

Unhealthy drinking can contribute to many problems. Stopping or reducing drinking can have many important benefits. Tick (✓) the ones that would apply to you.

| | | |
|---|---|---|
| Improved family relationship Reduction in domestic violence ☐ | Improved financial situation ☐ | Better physical and mental health ☐ |
| Having an active personal and social life ☐ | Regaining lost social status ☐ | Reduced mental health problems for other family members ☐ |
| Reduced risk of physical injury ☐ | Developing interest in new enjoyable activities ☐ | Improvement in work performance, less absenteeism ☐ |

## When Stopping Is Advisable

Stopping is recommended in the following situations.
(Tick (✓) as appropriate)

- Physical health problems which are the result of drinking. ☐
- Health problems where drinking can be dangerous, e.g. liver problems, high blood pressure or diabetes. ☐
- Mental health problems like anxiety and depression. ☐
- Long history of harmful drinking. ☐
- Older adults. ☐
- Addiction. ☐
- those having less support from family and friends. ☐
- Those who have tried and failed to control their drinking. ☐



## What You Can Do To Control Your Drinking

Some of the skills that a patient aiming for controlled drinking can sue are.

### Before drinking — What to do

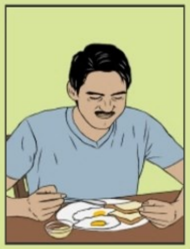

- Eat something first.
- Go out later.
- Take less money.
- Don't drink in places where you have previously drunk heavily.
- Take alcohol-free drinks with you to parties, friends' houses, et.

### While drinking — What to drink

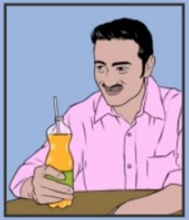

- Drink a long soft drink first.
- Drink pints vs large beers; singles vs doubles; ordinary vs strong beers.
- Drink more diluted drinks: spirits with mixers as opposed to neat, shandy (beer plus lemonade) vs all-beer, half-and-half or alcohol-free and ordinary beer or lager.
- Drink low or no-alcohol beers and wines.
- Alternate between non-alcoholic and alcoholic drinks.

### While drinking — How to drink

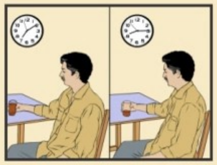

- Family Problems:
- Family tension
- Quarreling and violence
- Missed family functions
- Family members may become anxious, depressed, and may drink excessively themselves
- Children may have behavior problems and perform poorly in school
- Financial problems and debts
- Social Difficulties:
- Conflict with colleagues Workplace accidents
- Frequent absence from work
- Repeated dismissals from work and long-term unemployment
- Arguments and fights
- Problems with the police
- Loss of friends

### A brief example of a controlled drinking plan:

Rules- What you want to achieve?

How you are going to achieve it?

# How to fight The Urge To Drink

Some people with drinking problems find the following techniques useful to fight the urge to drink. You could try them too.

Get involved in non-drinking activities: for example walking, gardening or other physical activities, reading, or try writing about your thoughts and feelings.

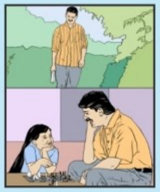

Go to a "safe" place: e.g. a non-drinking friend's house, a temple/ church/ mosque or a library.

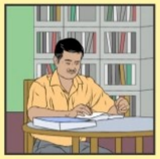

Talk about the urge to drink with family or a friend and get some help and support from them.

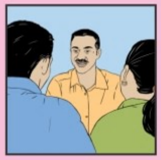

Remind your that the urge is time limited.

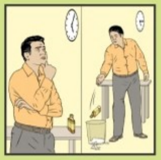

Face the urge and resist it.

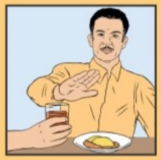

Consume other things like your favorite food or a non-alcoholic drink when you get the urge.

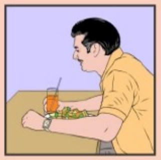

Prepare a small reminder card and keep it in your pocket. On it, you can write your reasons for controlling/stopping drinking and the problems you have faced because of your drinking.

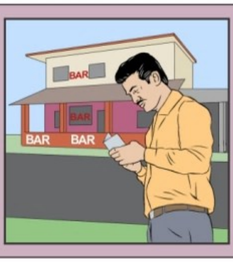




# Some Strategies to Reduce or Stop Drinking

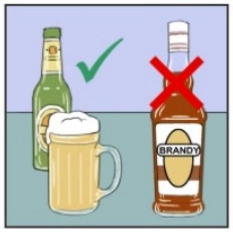


Not all brands of alcohol are the same strength. If you don't want to stop drinking, then cons/der drinking drinks which contain less alcohol e.g. beer Instead of brandy.

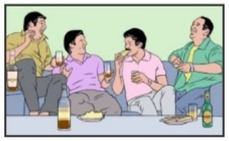

If you are drinking with others, drink at the same pace as the lowest-drinking person.

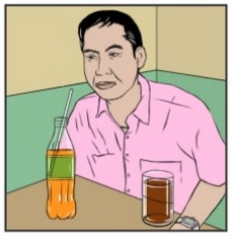

Pace your drinking. Plan how long you will be out and how many drinks you will have and stick to your plan; sip, don't gulp; out your glass down between sips; drink more slowly; alternate alcoholic and non-alcoholic drinks.

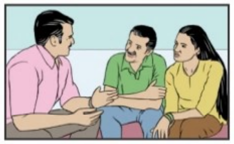

Involve your family or a friend. They can help you decide on a goal with regards to drinking and help keep track of progress.

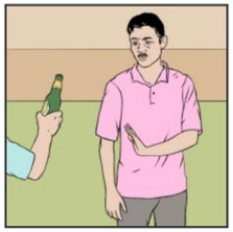

Know how to refuse a drink. Decide for yourself when and how much you want to drink.

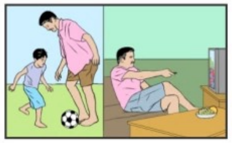

Find other things you can do Instead of drinking

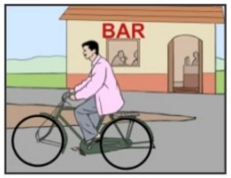


Avoid high-risk drinking situations like going to the bar or meeting with others who drink (or who drink heavily, if you are aiming to control your drinking).

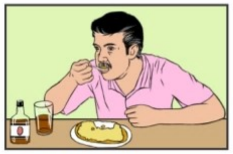

Eat before and while drinking.

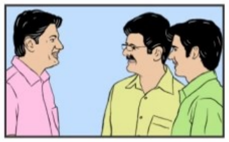

talk through your problems and worries. stress and worries can influence how much one drinks. Talking about them can be a good first step to dealing with problems related to alcohol.

# Solving Life Problems

from time to time, all of us struggle to handle life problems, you might come across such problems yourself. Sometimes your inability to handle these problems can be due to your alcohol related problems,

**You could use the following 5 steps to try and tackle such problems effectively.**

Define the problem clearly, be specific and try to narrow in on what exactly is the problem.

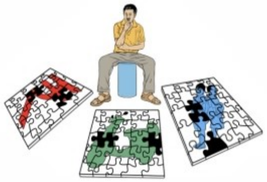

Try to find different solutions to the problem.

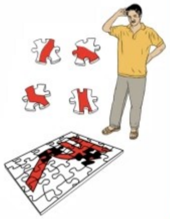

Think through each solution and check for advantages of the solution. Choose the solution that promises the best result.

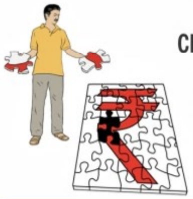

Plan to carry out the solution. Practice applying the solution.

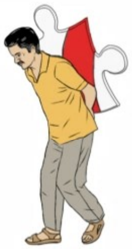

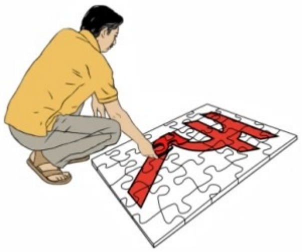

Use a time limit to test the solution. Assess the outcome.



# Solving Problems Woksheet

Use this page to try out the steps for your problems:

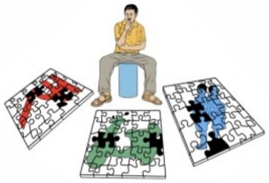

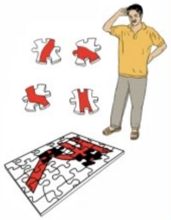

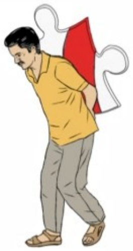

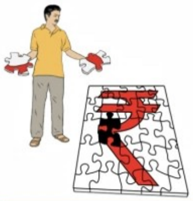

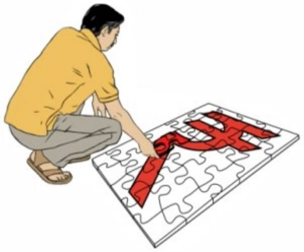

# Saying 'No" to Drinks

It is useful to be able to say 'NO' when offered a drink. If you have stopped or reduced your drinking it is important to be prepared to handle such situations by using certain simple techniques.

Attending a party (either alone or with friends or family).

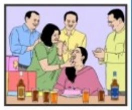

Your visit friends or family members who drink.

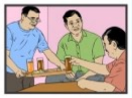

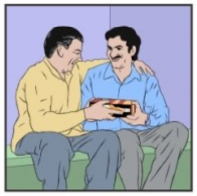

Bottle of Alcohol or drink offered as gift on specific occasions.

Friends or family members who drink visiting you.

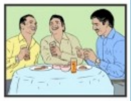

Visit to a bar or a restaurant where alcohol is being served.

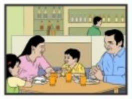

**Things to rmember**

- When a friend offers you a drink saying 'NO' to the drink does not mean 'NO' to a friendship.
- Maintain eye contact with the person who offers you the drink when you say 'NO'.
- Speak firmly and convincingly. Your expression and tone should indicate that you are serious.
- You could use the following responses if somebody offers a drink:

**You could use the following responses if somebody offers a drink:**

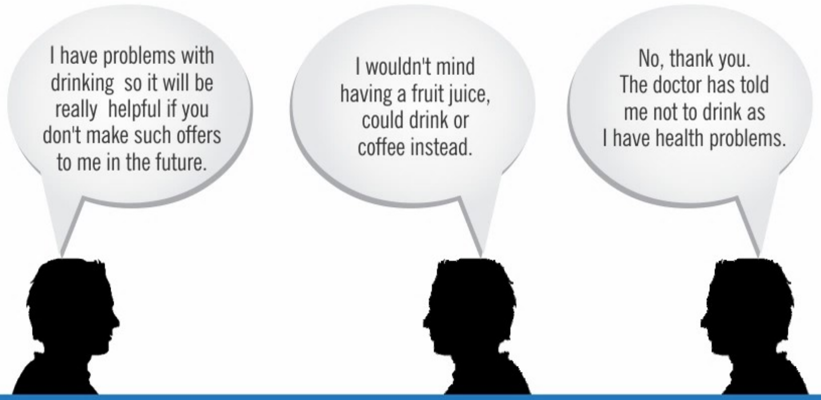






# Things , I Will do

**End of Treatment Form**

Date:____________

To remain healthy I will do following things

| I will stop continue doing following things | I will stop doing following things |
|---|---|
| | |
| | |
| | |
| | |
| | |
